# TEM8 functions as a receptor for uPA and mediates uPA-stimulated EGFR phosphorylation

**DOI:** 10.1186/s12964-018-0272-8

**Published:** 2018-09-21

**Authors:** Lian-Cheng Zhang, Yong Shao, Li-Hua Gao, Jin Liu, Yong-Yi Xi, Yin Xu, Chutse Wu, Wei Chen, Hui-Peng Chen, You-Liang Wang, Hai-Feng Duan, Xian-Wen Hu

**Affiliations:** 1 Laboratory of Cell Engineering, Beijing Institute of Biotechnology (BIB), No. 20, Dongdajie Street, Fengtai District, Beijing, 100071 China; 2 Key Laboratory of Experimental Hematology, Beijing Institute of Radiation Medicine (BIRM), No. 27, Taiping Road, Haidian District, Beijing, 100850 China; 3 Department of Operational Medicine, Tianjin Institute of Environmental and Operational Medicine, Tianjin, 300050 China

**Keywords:** EGFR, Phosphorylation, Receptor, TEM8, uPA

## Abstract

**Background:**

TEM8 is a cell membrane protein predominantly expressed in tumor endothelium, which serves as a receptor for the protective antigen (PA) of anthrax toxin. However, the physiological ligands for TEM8 remain unknown.

**Results:**

Here we identified uPA as an interacting partner of TEM8. Binding of uPA stimulated the phosphorylation of TEM8 and augmented phosphorylation of EGFR and ERK1/2. Finally, TEM8-Fc, a recombinant fusion protein comprising the extracellular domain of human TEM8 linked to the Fc portion of human IgG1, efficiently abrogated the interaction between uPA and TEM8, blocked uPA-induced migration of HepG2 cells in vitro and inhibited the growth and metastasis of human MCF-7 xenografts in vivo. uPA, TEM8 and EGFR overexpression and ERK1/2 phosphorylation were found co-located on frozen cancer tissue sections.

**Conclusions:**

Taken together, our data provide evidence that TEM8 is a novel receptor for uPA, which may play a significant role in the regulation of tumor growth and metastasis.

**Electronic supplementary material:**

The online version of this article (10.1186/s12964-018-0272-8) contains supplementary material, which is available to authorized users.

## Background

Tumor endothelial marker 8 (TEM8), a type I membrane protein, was originally identified by virtue of its elevated expression during tumor angiogenesis [1], and subsequently discovered to be a receptor for anthrax toxin, hence its official name, anthrax toxin receptor 1 (ANTXR1) [2]. TEM8 shares interesting similarities with integrins; however, its exact molecular function is unknown. The current knowledge of, and interest in, TEM8 and CMG2 (capillary morphogenesis gene 2) [3], another anthrax toxin receptor, largely stems from its role in infection by *B. anthracis*.

Since the initial reports showing that TEM8 expression is elevated during the process of angiogenesis, it has been considered as a potential target for anti-angiogenic therapy. Multiple functions have been reported for TEM8 in tumor angiogenesis: TEM8 is involved in the interaction of the cells with ECM proteins and is a component of signaling pathways regulating endothelial cell adhesion, migration, proliferation and angiogenesis. In a recent study, we demonstrated that a recombinant fusion protein comprising the extracellular domain of human TEM8 linked to the Fc portion of human IgG1 (TEM8-Fc) has potent anti-tumor properties [4]. More recently, antibodies against the TEM8 extracellular domain have been developed, which inhibited tumor-induced angiogenesis and displayed broad antitumor activity [5]. Taken together, these findings provide further support to the notion that targeting TEM8 may be a promising anti-tumor strategy.

The most well-known function of TEM8 is its role as a cell surface receptor for anthrax toxin PA. TEM8 mediates the entry of anthrax toxin into the cells of host organisms, and its role in the pathogenic process has been well described. However, in the absence of toxins, the physiological function of TEM8 is poorly understood. In recent years, it has been shown that TEM8 not only binds to ECM proteins, but also interacts with the cytoskeletal protein, actin. The extracellular domain of TEM8 interacts with collagen I, gelatin and collagen VI [6–8]. The cytosolic tail of TEM8 interacts directly with the actin cytoskeleton, and overexpression of TEM8 facilitates cell spreading by mediating signals from matrix ligands to the intracellular cytoskeleton [9–12]. Therefore, in a similar manner to the integrins, TEM8 is able to mediate inside-out (actin to the vWA domain) and outside-in (PA or physiological ligand to actin) signaling [13].

It has been reported that the intracellular domains of the TEM8 cell receptor interact with intracellular signaling molecules, including Src [14]. The Src family of kinases is involved in multiple signaling pathways including invasion, proliferation and survival. The anthrax toxin triggers Src-like kinase-mediated phosphorylation of the TEM8 intracellular domain, which is required for efficient uptake of anthrax toxin [14]. Therefore, it is of interest to understand whether similar interactions and signaling pathways occur in endothelial cells during angiogenesis, and whether TEM8 mediates the entry of its physiological ligand into cells using a mechanism similar to the entry of anthrax toxin. To some extent, TEM8 can be regarded as a single chain of integrin [13]. Integrins form a functional complex with EGFR and uPAR to mediate cell migration and angiogenesis. Similarly, TEM8 may play a role in cell migration and angiogenesis [15]. These interactions may provide a mechanism to regulate ECM protein interactions and/or cell surface complex-induced signal transduction. In order to clarify the precise physiological function of TEM8 at the cellular level, it is of paramount importance to identify its natural ligands.

In this study, we used TEM8-Fc as bait to search for the natural interacting partners of TEM8, in order to further elucidate the molecular basis for its anticancer activity. Our data show that uPA is a physiological ligand for TEM8. This finding is important for understanding the role of TEM8 in tumor growth and metastasis, and also provides insight to explain the uPAR-independent functions of uPA in several physiological and pathological conditions.

## Methods

### Cells and siRNA

HEK293 cells, CHO-K1 (CHO) cells, MCF7 cells and HepG2 cells were purchased from ATCC and maintained in DMEM/F12 (1:1) (Hyclone, Logan, UT, USA) supplemented with 10% fetal bovine serum, penicillin (100 units/ml) and streptomycin (100 μg/ml), unless indicated otherwise. The cells were cultured in 5% CO_2_ and 95% humidity at 37 °C.

TEM8 and uPAR knockdown was performed using synthetic RNA duplexes purchased from Santa Cruz Biotechnology, Inc. (Dallas, Texas, USA).

The suppliers of antibodies and reagents were listed in Additional file 1.

### Antibodies and reagents

The protein A Sepharose 4 Fast Flow affinity column was purchased from Amersham Biosciences, Uppsala, Sweden; Streamline™ rProtein A was purchased from GE Healthcare, Uppsala, Sweden; Herceptin was obtained from Roche, Grenzach-Wyhlen, Germany; PA was from Merck, Darmstadt, Germany; protease inhibitor cocktail, type I collagen and chromogenic urokinase substrate S-2444 were purchased from Sigma, St Louis, MO, USA; Lipofectamine™ 2000 reagent was from Gibco BRL, Grand Island, NY, USA; rabbit IgG and protein A/G-Sepharose were from Santa Cruz Biotechnology, Inc., Santa Cruz, CA, USA; antibodies recognizing RFP and GFP were purchased from MBL, Nagoya, Japan; anti-uPA, anti-uPAR and anti-TEM8 (ab15724) antibodies were from Abcam Ltd., Cambridge, UK; anti-phospho-EGFR (Tyr845), anti-phospho-EGFR (Tyr1173), anti-phospho- ERK1/2 and anti-phosphotyrosine (p-Tyr100) antibodies were from Cell Signaling Technology, Inc., Beverly, MA, USA; horseradish peroxidase-conjugated secondary antibodies (anti-rabbit IgG, anti-goat IgG, anti-mouse IgG and anti-human IgG) and anti-actin were from Beijing Zhongshan Jinqiao Biotechnology Ltd., Beijing, China.

### Analysis of binding of TEM8 to recombinant uPA by ELISA

HMW-tcuPA, the full-length two-chain high molecular weight urokinase-type plasminogen activator; HMW-scuPA, the single chain high molecular weight zymogen; LMW-uPA, the low molecular weight uPA enzyme.

Interaction of the TEM8-Fc fusion protein with uPA was examined by direct ELISA. There are three forms of uPA, namely the single chain zymogen, termed Pro-uPA (also known as high molecular weight single-chain urokinase-type plasminogen activator, HMW-scuPA), the full-length two-chain uPA enzyme (also known as high molecular weight two-chain urokinase-type plasminogen activator, HMW-tcuPA), and the low molecular weight urokinase-type plasminogen activator (LMW-uPA). To do this, 96-well plates were coated with 0.1 μg of recombinant PA, HMW-scuPA, commercially available HMW-tcuPA (National Institute for Biological Standards and Control, UK), or LMW-uPA in Tris-buffered saline (TBS) for 16 h at 4 °C. The plates were washed three times with TBST (0.05% Tween-20 in TBS), and blocked with 100 μl/well of blocking buffer (3% BSA in TBS) for 1 h at room temperature. After three washes, 0–250 ng of purified TEM8-Fc or Herceptin in 100 μl diluting buffer (3% BSA, 0.05% Tween-20 in TBS) in the presence of 2 mM MnCl_2,_ was added to the wells and incubated at 37 °C for 1 h. Following three washes, the samples were incubated with 100 μl/well of diluted HRP conjugated goat anti-human IgG (1:20,000) for 1 h at 37 °C. The plates were washed and 100 μl of developing solution (3,3′,5,5’-Tetramethylbenzidine/H_2_O_2_) was added. After 15 min, stop solution (2 M H_2_SO_4_) was added and the plates were analyzed on a microplate reader at a wavelength of 450 nm. The results were calculated as mean absorbance (A_450nm_) of triplicate wells for each sample.

### Affinity chromatography and MS/MS

The fusion protein consisting of the N-terminal 1–227 amino acid residues of human TEM8 linked to the Fc portion of human IgG1 (TEM8-Fc) was produced, as described previously [4]. Human hepatoma tissue was homogenized in RIPA buffer and centrifuged. The supernatant was applied to a protein A Sepharose 4 Fast Flow affinity column to remove endogenous IgG proteins and was transferred to a protein A Sepharose 4 Fast Flow affinity column pre-conjugated with the TEM8-Fc. Bound proteins were eluted and separated by SDS-PAGE, and then stained by Coomassie. A single distinct band of approximately 50 kDa was observed. This band was excised from the gel, incubated with trypsin, and then analyzed by nano LC-MS/MS using CapLC liquid chromatography coupled to a Q-TOF Ultima fusion quadrupole time-of-flight mass spectrometer (Waters, Milford, MA, USA). MS/MS fragment ion spectra were searched against the NCBI nr protein sequence database using the MASCOT database search engine (Matrix Science, London, UK).

### Fluorescence resonance energy transfer (FRET) measurements

FRET vector construction: The region of the TEM8 gene encoding the extracellular domain was subcloned into the pcDNA3.1/hygro plasmid (Invitrogen) between the *Bam*HI and *Eco*RI restriction sites. RFP was fused to the C-terminal end of *TEM8*. The uPA gene sequence was cut into three parts: the EGF-like domain, the kringle domain and the LMW domain. Since the LMW domain is large and constitutes two-thirds of the entire uPA sequence, the LMW domain was split into two parts, namely the CAT portion and the uPACT portion, according to the position of the disulfide bond. The EGF-like domain, the kringle domain, the LMW domain, the CAT portion and the uPACT portion were subcloned into the plasmid pcDNA3.1 (Invitrogen). Anthrax toxin PA domain 4 was also cloned into pcDNA3.1. EGFP was fused to the C-terminal end of PA domain 4, and truncated uPA proteins. All constructs were verified by sequencing the entire coding region. The resulting constructs were designated TEM8-RFP, EGF-like-EGFP, kringle-EGFP, LMW-EGFP, CAT-EGFP, uPACT-EGFP and PA-EGFP.

Imaging with confocal FRET microscopy: Lipofectamine™ 2000 reagent was used to introduce the FRET vectors into human HEK293 cells, and the transfected cells were selected by G418 and hygromycin. Cells forming stable colonies under selection pressure were prepared for imaging analysis. Confocal images were acquired at the Optical Imaging Facility of the National Center of Biomedical Analysis (NCBA) using a Zeiss Laser Scanning Microscope (LSM) 510 META confocal microscope (Carl Zeiss, Inc., Thornwood, NY) equipped with Laser Scanning Microscope LSM 510 Release version 4.2. All images were captured with an oil immersion objective using the following fluorescence filters: GFP filter (Df; excitation 488 nm, dichroic mirror 560 DCLPXR, emission HQ 515/30), RFP filter (Af; excitation 543 nm, dichroic mirror 650 DCLPXR, emission HQ590/70) and FRET filter (Ff: excitation 488 nm, dichroic mirror 650 DCLPXR, emission HQ590/70) to analyze protein interactions [16]. To calculate N_FRET_, a normalized FRET signal, we used the following equation:

$$ {N}_{FRET}=\frac{Ff- Df\times \left( Fd/ Dd\right)- Af\times \left( Fa/ Aa\right)}{\sqrt{Df\times Af}}, $$

where Ff, Df, and Af are intensities in each region of interest (ROI) under FRET, GFP, and RFP filter sets, respectively. Fd/Dd represents the amount of crosstalk from the donor signal into the FRET channel, and Fa/Aa represents the amount of crosstalk from the acceptor signal into the FRET channel. There were no bleed-through signals from GFP under RFP filter sets, and vice versa. The values for bleed-through varied with different imaging systems. The normal values for Fd/Dd and Fa/Aa for the system used in the present study were 22.23% and 79.41%, respectively, which were determined by analyzing images of cells expressing only GFP or RFP and quantifying the relative intensity ratio under the FRET/GFP or FRET/RFP filter sets. Pixel-by-pixel corrected FRET (FRETc) images were created using only the calculated dividend values from the above eq. A special color reference table was used to indicate the value of the corresponding pixels. Blue shades were negative, with bright blue representing the most negative numbers (only visible when the corresponding option setting was not checked, and not available for FRETn and N_FRET_ images), black was equivalent to pixels with zero intensity, and positive numbers were shown with increasing value in the following colors: cyan, green, yellow, red, white.

### Kinetic analysis with surface Plasmon resonance (SPR)

SPR studies were performed using a BIAcore 2000™ biosensor system (BIAcore AB, Uppsala, Sweden). For immobilization, purified TEM8-Fc was diluted in sodium acetate buffer of pH 5.0, 4.5 or 4.0. Following activation of the carboxymethylated dextran surface with N-hydroxysuccinimide/ 1-ethyl-3-(3-dimethyl ami-nopropyl) carbodiimide, the proteins were injected onto a CM5 sensor chip. A series of concentrations of HMW-scuPA, HMW-tcuPA, LMW-uPA and PA in PBS, with or without MnCl_2,_ were then passed over the sensor chip at a flow rate of 30 μl/min. Equivalent concentrations of each analyte were injected over the Herceptin surfaces as background controls. The sensor chip surface was regenerated with NaOH after each injection. Association and dissociation rate constants were calculated by nonlinear fitting of the primary sensorgram data using BIA evaluation 3.0 software (BIAcore AB).

### Functional assay of fibrinolytic activities for TEM8-bound uPA

Fibrinolytic activity of TEM8-bound uPA was assayed on human plasminogen-rich fibrinogen agarose plates using a procedure modified from Binder et al. [17]. Standardization of the plasminogen-rich fibrinogen/agarose plates was performed with serial dilutions of the international standard for HMW-tcuPA (National Institute for Biological Standards and Control, UK), and the standard curve was plotted as the log concentration of HMW-tcuPA versus the diameter of the lysis zone. Samples were applied in the same way as the standards; 5 μl of 50 IU/ml of the HMW-tcuPA standard plus 5 μl of the serial dilutions of TEM8-Fc were included in each well. As controls, fibrinolytic activities were also measured with HMW-tcuPA standard plus anti-uPA rabbit polyclonal antibody, or HMW-tcuPA standard plus anti-ATF mAb. The anti-ATF antibody only binds to recombinant ATF-6 × His expressed in Chinese hamster ovary cells (CHO) and shows no cross-reactivity with recombinant LMW-uPA. Replicate wells were analyzed for each sample.

### Chromogenic activity assay

The activity of the binding of HMW-tcuPA to CHO cells was determined by a spectrophotometric assay using the chromogenic uPA substrate, S-2444. In brief, CHO cells were cultured until they reached approximately 90% confluence. The cells were fixed with 95% ethanol, and treated with glycine-HCl buffer (pH 3.0) to remove endogenous uPA. After blocked with the blocking solution, the cells were incubated with increasing concentrations (125 nM to 2000 nM) of human HMW-tcuPA or HMW-tcuPA pre-incubated with TEM8-Fc for 20 min (final concentration of 2000 nM). After the CHO cells were washed to remove unbound HMW-tcuPA, 50 μl aprotinin (100 KIU/ml diluted in TBST, Trasylol, Bayer) and 50 μl of S-2444 (1.5 mg/ml dissolved in TBST) were added to the wells. The absorbance at 405 nm (A_405nm_) was measured using a microplate reader (Bio-Rad Model 550, Hercules, CA, USA), and HMW-tcuPA activity was determined by calculating the change in absorbance with incubation time (ΔA_405nm/_min). Meanwhile, the binding of LMW-uPA to HepG2 cells was also determined by a chromogenic activity assay. To verify that TEM8-Fc specifically blocks the binding of LMW-uPA to HepG2 cells, the Fc-containing, humanized antibody Herceptin was used as a negative control.

### Immunofluorescence

Dual-color immunofluorescence was performed to determine the cellular localization of TEM8 and EGFR. Hepatoma HepG2 cells were serum-starved overnight and stimulated by LMW-uPA for 30 min, in the presence or absence of pretreatment with TEM8-Fc. The cells were fixed and stained with anti-TEM8 (Abcam Ltd., Cambridge, UK) and anti-EGFR (Cell Signaling Technology, Inc., Beverly, MA, USA) antibodies. TEM8 was detected with a PE-conjugated anti-rabbit antibody (Beijing Zhongshan Jinqiao Biotechnology Ltd., Beijing, China) and EGFR was detected using a FITC-conjugated anti-mouse antibody (Beijing Zhongshan Jinqiao Biotechnology Ltd., Beijing, China). Cells were visualized by confocal microscopy.

### Human RTK and EGFR phosphorylation antibody Array screening

HepG2 cells were incubated in serum-free media overnight, and then treated with LMW-uPA and/or TEM8-Fc for 15 min. The subsequent steps were performed according to the protocol obtained from the manufacturer of the Antibody Arrays. Signal intensities were analyzed using the Gene Pix array 4000B scanner (Molecular Dynamics Inc., Sunnyvale, CA, USA). Relative phosphorylation levels were calculated using the Gene Pix pro software (Molecular Dynamics Inc. Sunnyvale, CA, USA). A biotinylated protein produces positive control signals, which can be used to identify the orientation of the chip and to normalize and compare the results in different wells.

## Results

### Identification of TEM8 interacting partners

**Fig. 1:**
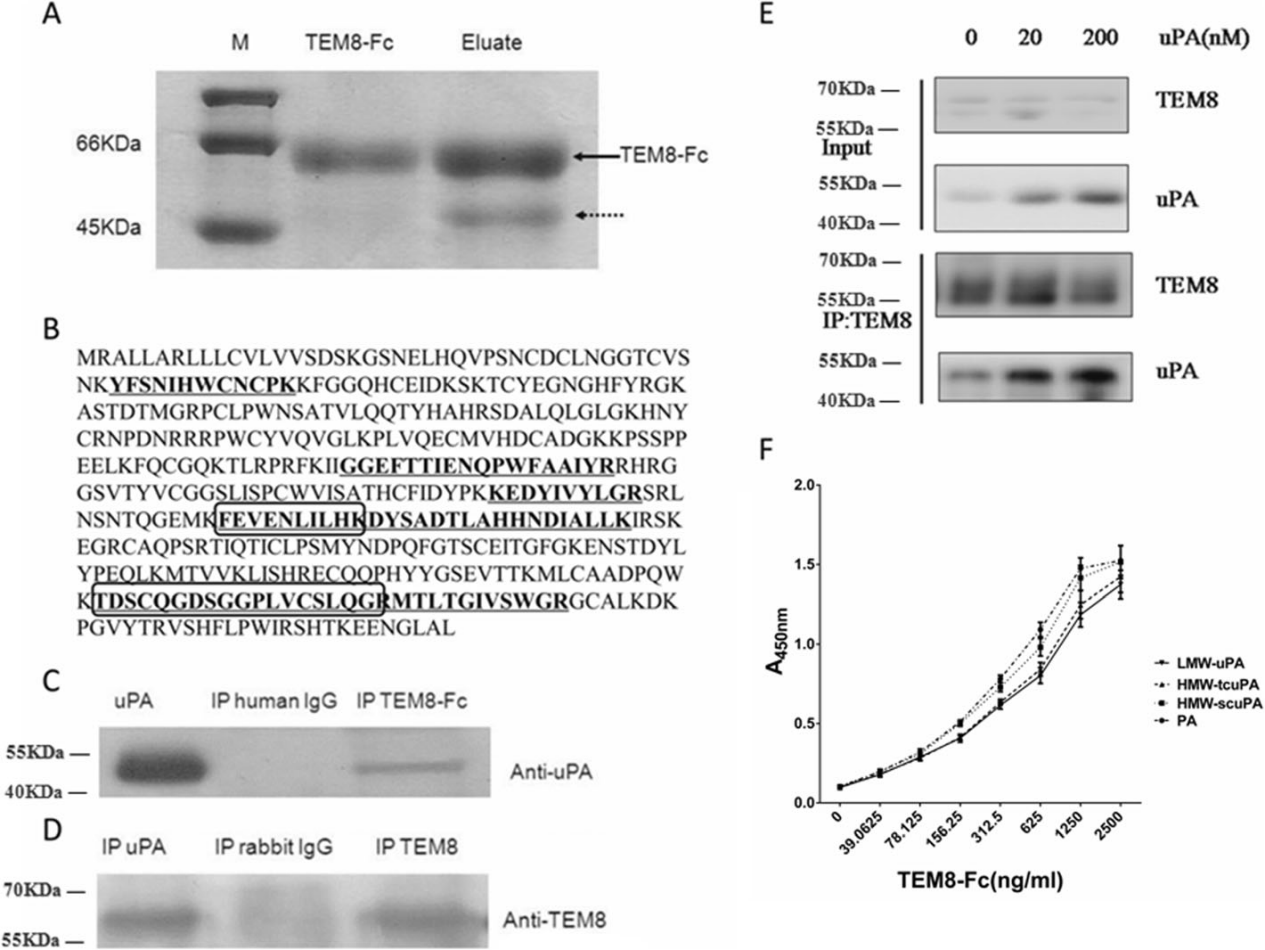
A 50 kDa protein, identified as uPA, associates with the extracellular domain of TEM8. **a** Human hepatoma tissue lysates were applied to a TEM8-Fc -conjugated Protein A Sepharose affinity column. Purified TEM8-Fc and the eluate were separated by 12% SDS-PAGE and stained with Coomassie brilliant blue. The dotted arrow indicates a TEM8-Fc -binding protein of approximately 50 kDa. **b** Sequence of the uPA protein. The amino acid residues of tryptic peptides identified by mass spectrometry are underlined or enclosed in boxes. **c** The hepatoma tissue homogenate was immunoprecipitated with TEM8-Fc or human IgG as a control, and binding to uPA was detected by immunoblotting with an anti-uPA antibody. **d** The hepatoma tissue homogenate was immunoprecipitated with anti-TEM8 or anti-uPA antibodies, or isotypic IgG as a control, followed by immunoblotting with an antibody recognizing TEM8. **e** The HepG2 cells were treated with uPA for 2 h followed by ultrasonication to harvest the cell lysate. The lysate was immmunoprecipitated by TEM8 and then detected by immunoblotting using TEM8 or uPA antibody**. f** The extent of binding of increasing concentrations of TEM8-Fc to the wells of microtiter plates coated with 0.1 μg of purified PA, HMW-scuPA, HMW-tcuPA or LMW-uPA in the presence of 2 mM MnCl_2_. The data are expressed as means of triplicate samples analyzed from three representative experiments

Using a TEM8-Fc -conjugated Protein A sepharose affinity chromatography, a protein band of approximately 50 kDa was detected that was capable of binding TEM8-Fc (Fig. 1a, as indicated by the dotted arrow). The 50 kDa band was analyzed by mass spectrometry, and seven tryptic peptides were identified with amino-acid sequences that were 100% identical to the sequence of certain portions of uPA (Additional file 1: Table S1 and Fig. 1b). To further confirm the interaction between TEM8 and uPA, immunoprecipitation and immunoblotting experiments were performed using anti-uPA, or anti-TEM8 antibodies. As shown in Fig. 1c and d, TEM8-Fc bound uPA. The results of the further experiments demonstrated that the interaction between TEM8-Fc and uPA showed a dose dependent effect (Fig. 1e).

### The region of uPA responsible for binding to TEM8 is distinct from the interaction domain for uPAR

As mentioned above, there are three forms of uPA, namely HMW-scuPA, HMW-tcuPA, and LMW-uPA. uPA is initially translated as pro-urokinase containing 431 amino acid residues. After excision of the signal peptide consisting of 20 amino acid residues at the front end, the secretory inactive single-chain high-molecular-weight urokinase, HMW-scuPA is formed. Then, under the action of plasmin and cathepsin, the peptide bond between Lys158 and Ile159 was cleaved, and HMW-scuPA is transformed into the double-chain HMW-tcuPA with high catalytic activity [18]. The Lys136-Lys137 peptide bond in HMW-tcuPA could also be hydrolyzed by plasmin, which make HMW-tcuPA further transform into LMW-uPA, and LMW-uPA catalytic activity is equivalent to that of HMW-tcuPA (Additional file 1: Figure S1). Additional file 1: Figure S2 shows the SDS-PAGE gel of LMW-uPA, HMW-scuPA, and HMW-tcuPA under reducing and non-reducing conditions. As we known, uPAR specifically binds the amino-terminal fragment of uPA [19]. Therefore, uPAR binds HMW-scuPA and HMW-tcuPA, but not LMW-uPA. To clarify whether the region within uPA that binds to TEM8 is the same region that interacts with uPAR, direct ELISA was used to measure the ability of TEM8 to interact with three different forms of recombinant uPA. The result showed TEM8-Fc could bind with high affinity to HMW-scuPA, HMW-tcuPA or LMW-uPA (Fig. 1f), indicating that the binding site of uPA to TEM8 is not located in the amino-terminal region, and is distinct from the region that binds uPAR.

**Fig. 2:**
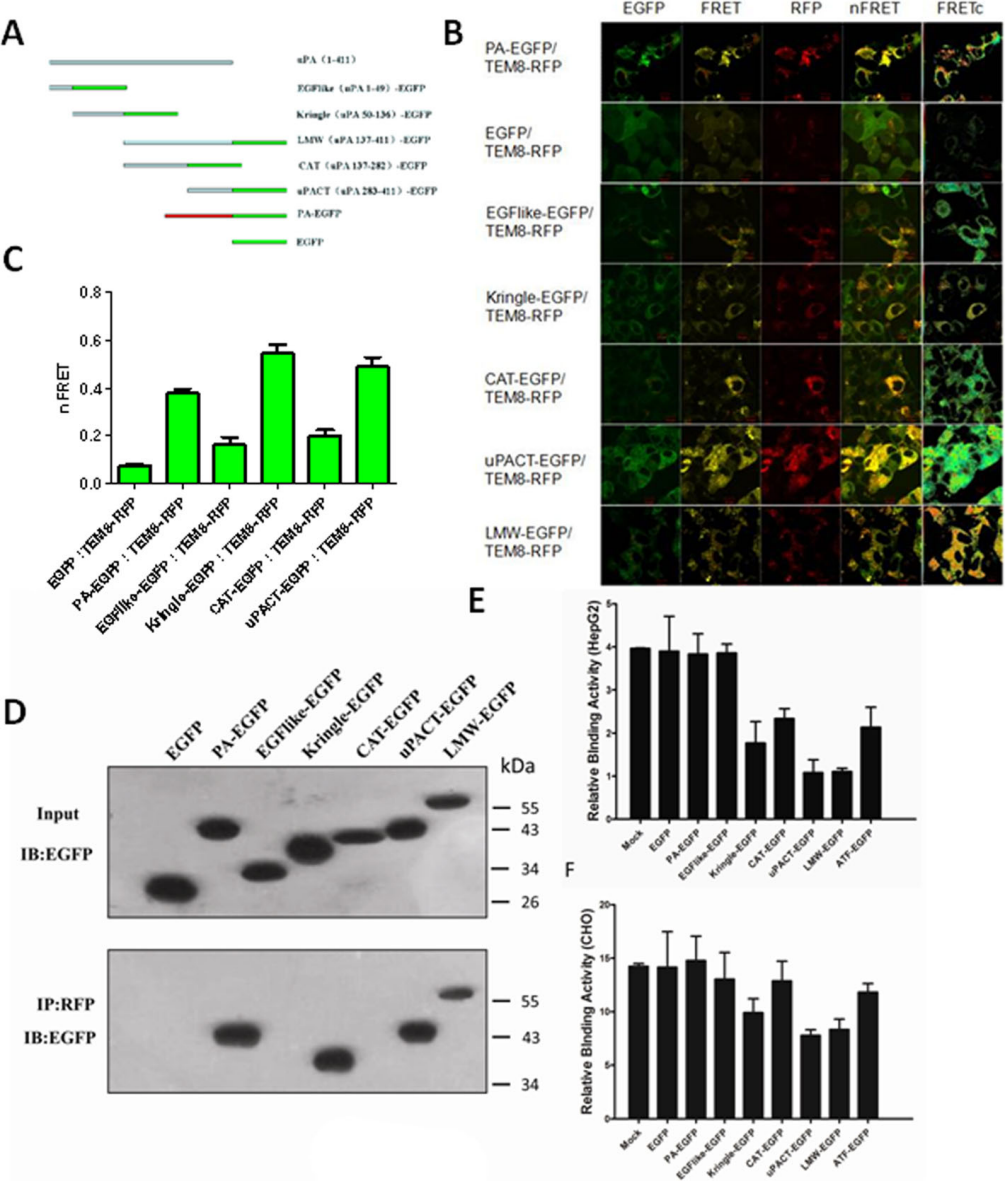
Identification of the region of uPA responsible for the interaction with TEM8. **a** Schematic representation of the full-length and truncated forms of uPA and full-length PA. EGFP was located at the C-terminus of the fusion proteins. **b** Representative laser scanning microscope FRET images. Paired constructs were co-transfected into HEK293 cells. Laser scanning microscope FRET images were captured from each of the three channels with the following excitation and emission wavelengths: EGFP (488/515 nm), FRET (488/590 nm) and RFP (543/590 nm). The labels on the top of each of the columns designate the three channels (green, FRET and red), then N_FRET_, and finally pixel-by-pixel corrected FRET (FRETc). The identity of the co-transfected constructs is indicated to the left of the panels. **c** The quantitative normalized FRET (N_FRET)_ values were calculated from laser scanning microscope FRET images. The N_FRET_ value is the average of regions of interest (ROIs) (*n* = 50–80) across three independent transfection experiments. ROIs were selected in areas that contained no protein aggregates. **d** Paired constructs were co-transfected into HEK293 cells. The cells were lysed, and protein extracts were subjected to SDS-PAGE and immunoblotting with anti-EGFP antibody (upper panel) or were immunoprecipitated with an anti-RFP antibody, and immunoblotted with an anti-EGFP antibody (lower panel). **e** & **f** Cells were cultured in 96-well cell culture plates and pre-treated with the several truncation mutants of uPA. The cells were fixed and treated with acidified buffer to remove endogenous uPA, then human HMW-tcuPA was added and the cell surface-based fibrinolytic activity was measured

To confirm the results and identify the region in uPA that interacts with TEM8, several truncation mutants of uPA were constructed, including the EGF-like domain (uPA aa 1–49), the kringle domain (uPA aa 50–136), the CAT portion (uPA aa 137–282), the uPACT portion (uPA aa 283–411), and LMW-uPA (uPA aa 137–411); all of these fragments were fused to EGFP (Fig. 2a). A truncated form of TEM8 (aa 1–227) was also fused to RFP. The anthrax toxin PA domain 4, fused to EGFP, was used as a positive control. RFP-tagged TEM8 was individually co-transfected into HEK293 cells with each of the EGFP-tagged constructs. FRET analysis was conducted to determine which portion of uPA interacted with the extracellular domain of TEM8 in living cells. Typical laser scanning microscope images are shown in Fig. 2b. The first three columns are images collected in the green, the FRET and the red channels, respectively. FRET filter images were used to calculate N_FRET_. The fourth column corresponds to the merged images and represents the overlap between the first three channels. The fifth column, which shows the FRETc images, is pseudocolor images displaying the variation in gray levels (pixel densities) as colors to facilitate a clearer indication of the extent of energy transfer and thus protein-protein interaction [20]. FRETc intensity, displayed as a color spectrum, ranges from the low to the high renormalization values according to a reference table, with blue (cold) indicating low values and red (hot) indicating high values. The nFRET values were normalized against green fluorescence (Fig. 2c), which meant that the nFRET value obtained from co-expressing PA-EGFP and TEM8-RFP was much larger than the value resulting from EGFP and TEM8-RFP co-expression. The same result was observed with kringle-EGFP, uPACT-EGFP and LMW-uPA-EGFP, suggesting that the kringle domain and the C-terminal domain of LMW-uPA are responsible for binding TEM8. However, the EGF-like-EGFP/TEM8-RFP and CAT-EGFP/TEM8-RFP cotransfections showed a significantly lower normalized nFRET value compared with either the PA-EGFP/TEM8-RFP, kringle-EGFP/TEM8-RFP, uPACT-EGFP/TEM8-RFP or LMW-EGFP/TEM8-RFP cotransfections, which indicated that the EGF-like domain and the N-terminal domain of LMW-uPA do not interact with TEM8, despite the FRET values being slightly higher than the negative control.

To further confirm the results of the FRET measurements, co-immunoprecipitation experiments were carried out on extracts from cells co-expressing TEM8-RFP and the various uPA truncation mutants (or PA), fused to EGFP. Protein extracts were immunoprecipitated with an anti-RFP antibody, followed by immunoblotting with an anti-GFP antibody. As expected, TEM8-RFP co-immunoprecipitated with PA-EGFP, kringle-EGFP, uPACT-EGFP and LMW-EGFP. In contrast, no significant binding was detected in the immunoprecipitates from cells expressing TEM8-RFP and EGFP, EGF-like-EGFP, or CAT-EGFP (Fig. 2d). Taken together, these data demonstrate that the kringle domain and the C- terminal domain of LMW-uPA are responsible for the interaction with TEM8.

Then we investigated the effects of the several truncation mutants of uPA on the binding of uPA to the HepG2 cell surfaces. We found that when the cells were pre-treated with kringle-EGFP, uPACT-EGFP or LMW-EGFP, the binding of uPA to the HepG2 cell surfaces were decreased or even abrogated (Fig. 2e), which provided further evidence that the kringle domain and the C-terminal domain of uPA are responsible for binding TEM8. And when pre-treated with ATF-EGFP and CAT-EGFP, the binding to the cell surfaces were slightly decreased than the negative control, which might be due to the part of the overlap binding sites of ATF-EGFP and CAT-EGFP. However, there had no significant differences between pre-treated with EGFP, EGF-like-EGFP, PA-EGFP and the negative control, and this is owing to no overlap binding sites exist. The same effects of the several truncation mutants of uPA were found on the binding of uPA to the CHO cell surfaces (Fig. 2f).

### Kinetics of the interaction between uPA and immobilized TEM8-fc

**Fig. 3:**
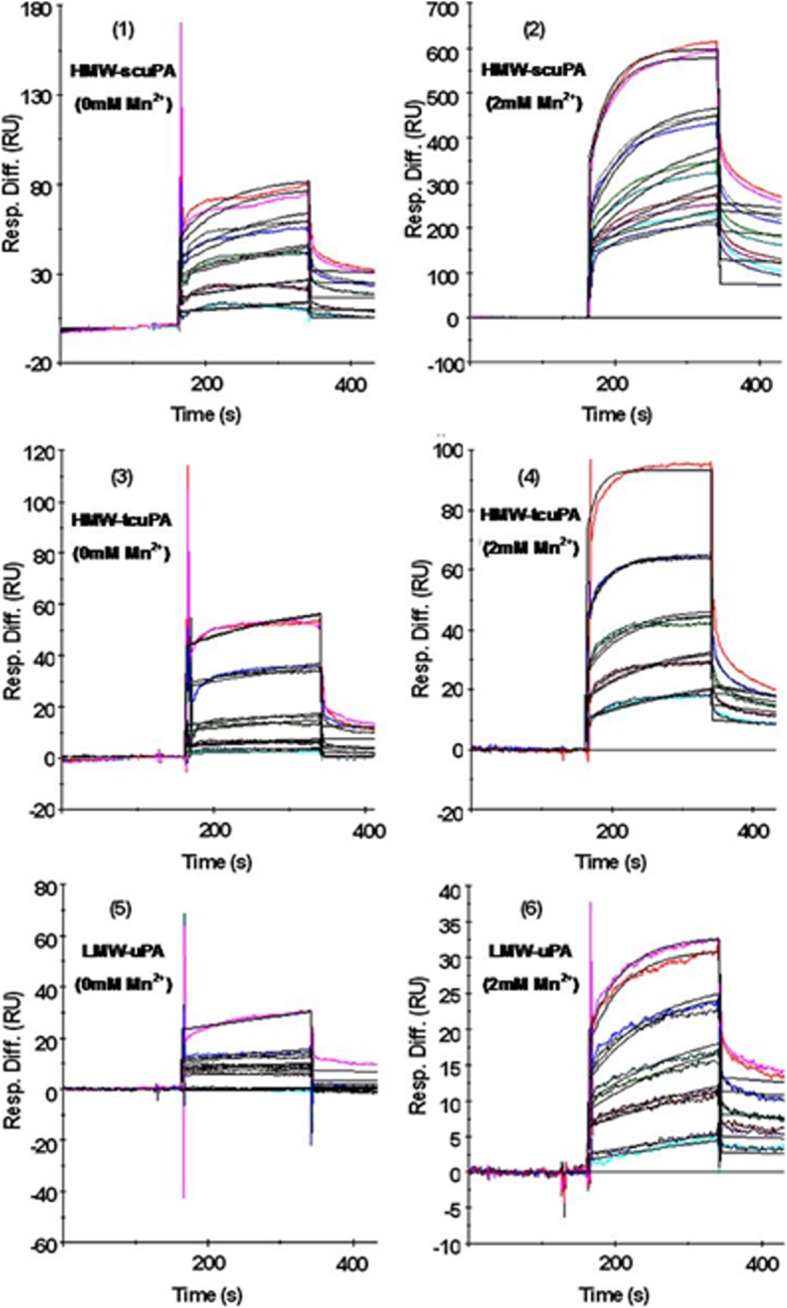
Association and dissociation of uPA from immobilized TEM8. A series of concentrations of HMW-scuPA, HMW-tcuPA or LMW-uPA (62.5, 125, 250, 500 and 1000 nM from bottom to top) were double-injected into a sensor chip pre-bound with approximately 5,000 resonance units of TEM8-Fc. All of the procedures were performed with running buffer in the absence of Mn^2+^ (Fig. 3, left side) or in the presence of 2 mM Mn^2+^ (Fig. 3, right side). Colored lines indicate the association and dissociation curves of the three forms of uPA with immobilized TEM8-Fc; black lines indicate the fitted curves

The surface plasmon resonance-based Biacore assay was used to investigate the kinetics of the interaction between TEM8 and HMW-scuPA, HMW-tcuPA, LMW-uPA or PA (the recombinant anthrax toxin PA domain 4). A series of concentrations of HMW-scuPA, HMW-tcuPA, LMW-uPA, and PA were passed over chips with immobilized recombinant TEM8-Fc or Herceptin. As shown in Additional file 1: Table S2 and Fig. 3, TEM8-Fc bound HMW-scuPA, HMW-tcuPA, LMW-uPA and PA, but not BSA. In the presence of Mn^2+^, HMW-tcuPA and LMW-uPA bound TEM8-Fc with a greater affinity, suggesting that the metal ion-dependent adhesion site (MIDAS) motif in TEM8 is involved in the uPA-TEM8 interaction. Interestingly, PA associated with, and dissociated from, TEM8-Fc more slowly than HMW-scuPA, HMW-tcuPA and LMW-uPA, which resulted in a relatively higher K_D_ value. Collectively, these data firmly support the hypothesis that uPA is a novel ligand for TEM8.

### The uPA-TEM8 interaction at the cell surface

**Fig. 4:**
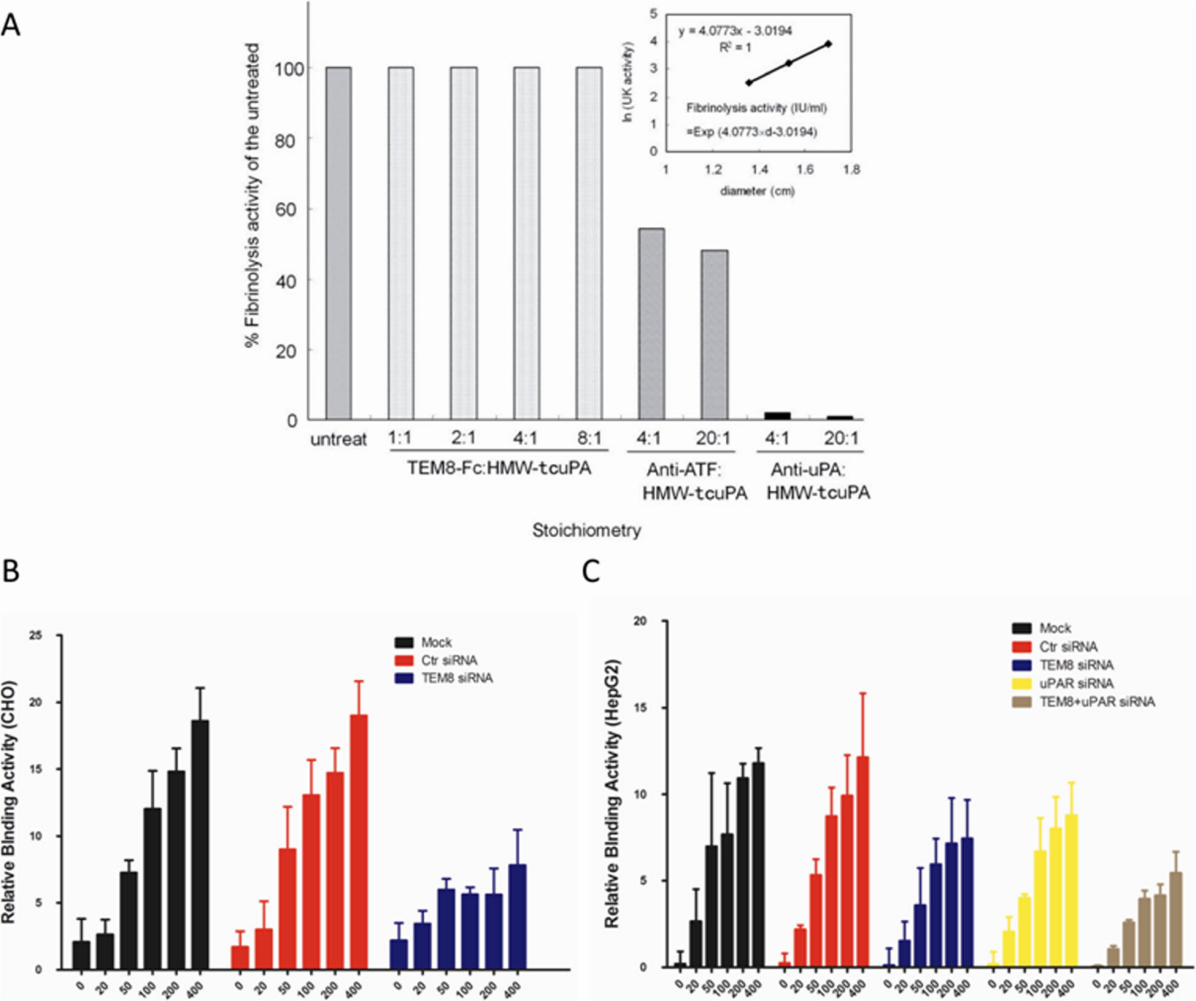
The uPA-TEM8 interaction at the cell surface. **a** The HMW-tcuPA protein was incubated with TEM8-Fc at different ratios, or with anti-uPA polyclonal antibody as a positive control. The fibrinolytic activity of TEM8-bound uPA was assayed on human plasminogen-rich fibrinogen agarose plates. The linearity of the plot, in the absence of TEM8-Fc, is shown in the insert. **b** & **c** Cells with wild-type or down-regulated TEM8 and/or uPAR were cultured in 96-well cell culture plates until they reached 90% confluence. The cells were fixed and treated with acidified buffer to remove endogenous uPA, then human HMW-tcuPA was added and cell surface-based fibrinolytic activity was measured

Since TEM8 binds LMW-uPA, a catalytically active form of uPA, it is of interest to establish whether the binding of uPA to TEM8 affects its enzymatic activity. As shown in Fig. 4a, the anti-uPA polyclonal antibody almost completely blocked the catalytic activity of HMW-tcuPA, and the anti-ATF monoclonal antibody decreased its activity by approximately 50%, probably due to spatial restrictions, as this monoclonal antibody does not bind the catalytic domain of uPA. However, TEM8-Fc had no influence on uPA catalytic activity in terms of the activation of plasminogen, even when the molar ratio of TEM8-Fc to HMW-tcuPA was as high as eight.

The binding of uPA to TEM8 at the cell surface was detected by two independent methods: binding of human HMW-tcuPA to CHO cells and HepG2 cells. TEM8 was knocked down by siRNAs in CHO cells. The cells with wild-type or down-regulated TEM8 were fixed and treated with acidified buffer to remove endogenous uPA, and then human HMW-tcuPA was added and cell surface-based fibrinolytic activity was measured. Human HMW-tcuPA could bind to the wild-type CHO cells, but not the CHO cells with down-regulated TEM8 (Fig. 4b). Since the binding of uPA to its receptor uPAR is rather species-specific, such that human uPA cannot bind to mouse uPAR, and vice versa [19], our results suggest that other molecules on the CHO cell surface can bind uPA. Previous studies have shown that CHO cells express TEM8 at a moderately high level (10^4^ molecules per cell) [21], and TEM8 is highly conserved across species (with 96% amino acid identity between the human and mouse proteins) [22]. Therefore, the molecule on the CHO cell surface that binds human HMW-tcuPA may be TEM8, because down-regulating TEM8 almost completely attenuated this binding. To further verify the interaction between uPA and TEM8 at the cell surfaces, human HMW-tcuPA was used to bind HepG2 cells again. TEM8 or uPAR were respectively knocked down by different siRNAs in HepG2 cells. As we have demonstrated, HepG2 hepatoma cells express a high level of TEM8. HMW-tcuPA binds to the wild-type HepG2 cell surfaces, and the knockdown of either TEM8 or uPAR could decrease the interaction of HMW-tcuPA with the cell surfaces, and moreover, the knockdown of TEM8 and uPAR at the same time could almost abrogate the interaction (Fig. 4c). All of the results above suggested that both TEM8 and uPAR were the receptors of uPA. In addition, human LMW-uPA, which lacks the ATF region (including the uPAR binding domain), was used to bind HepG2 cells in the other experiment. LMW-uPA could also bind the surfaces of HepG2 cells, and this interaction could be blocked by TEM8-Fc (Additional file 1: Figure S3). This provided further evidence that LMW-uPA interacts with TEM8, but not uPAR.

### TEM8 phosphorylation stimulated by uPA

**Fig. 5:**
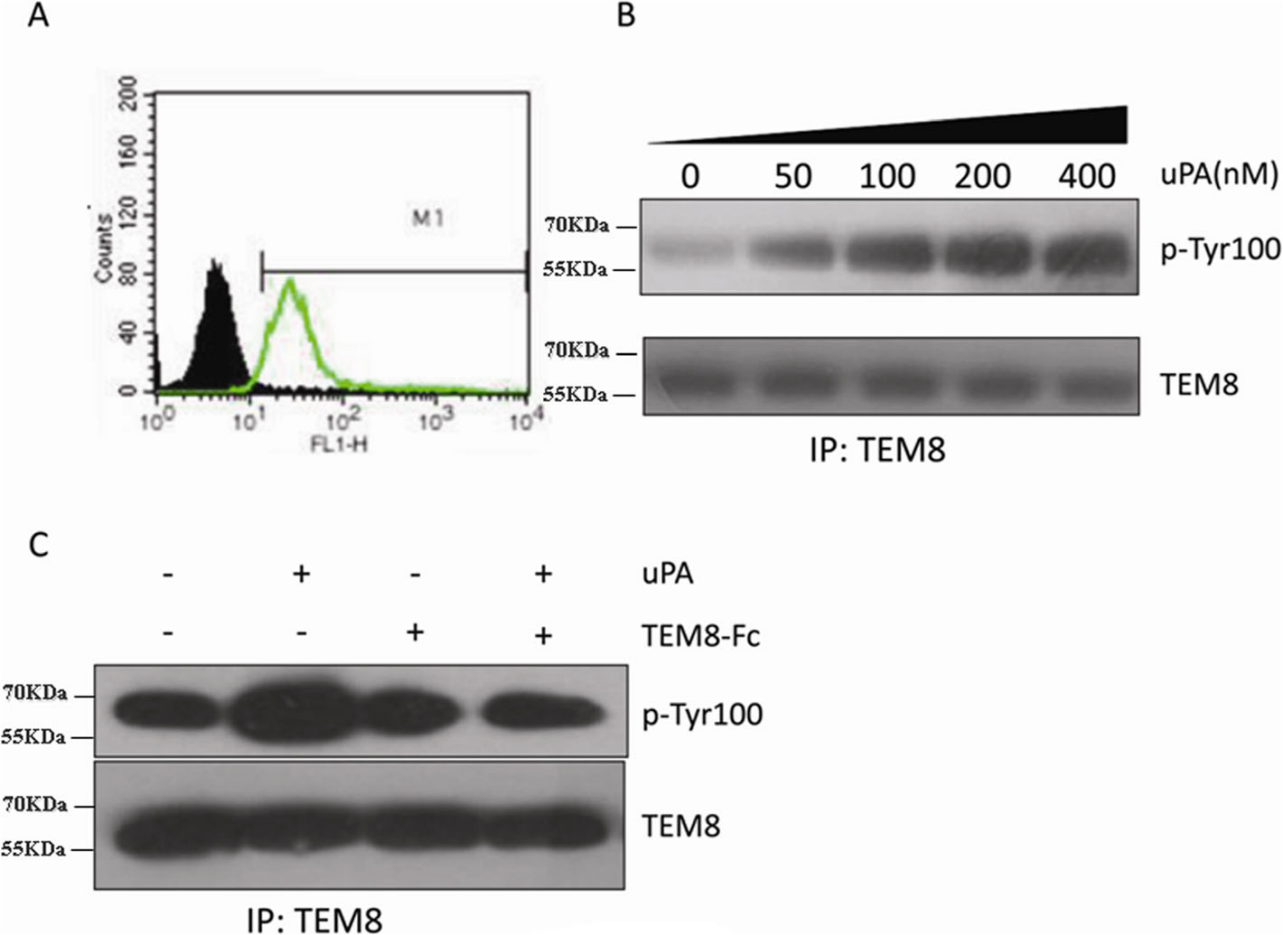
Stimulation of TEM8 phosphorylation by LMW-uPA. **a** HepG2 cells were incubated with (green, empty) or without (black, solid) an anti-TEM8 antibody, followed by detection with a FITC-labeled anti-IgG secondary antibody, measured by flow cytometry. **b** HepG2 hepatoma cells were serum-starved overnight and treated with LMW-uPA at the indicated doses. Cell lysates were immunoprecipitated with an anti-TEM8 antibody, followed by anti-p-Tyr100 immunoblotting. **c** HepG2 hepatoma cells were serum-starved overnight, pretreated with TEM8-Fc, and then stimulated with LMW-uPA. Cell lysates were immunoprecipitated with an anti-TEM8 antibody, followed by anti-p-Tyr100 immunoblotting

TEM8 is expressed by several human tumor cell lines, including HepG2 hepatoma cells (Fig. 5a). The cytoplasmic tail of TEM8 contains several potential phosphorylation sites, which prompted us to investigate whether TEM8 binding to uPA results in TEM8 phosphorylation. To this end, we investigated whether TEM8 tyrosine phosphorylation occurred when HepG2 cells, starved overnight, were stimulated with LMW-uPA. As shown in Fig. 5b, LMW-uPA treatment led to a dose-dependent phosphorylation of TEM8. Additional experiments showed that LMW-uPA-stimulated TEM8 phosphorylation could be reduced to pre-stimulated levels by TEM8-Fc (Fig. 5c).

### EGFR phosphorylation inducted by uPA

**Fig. 6:**
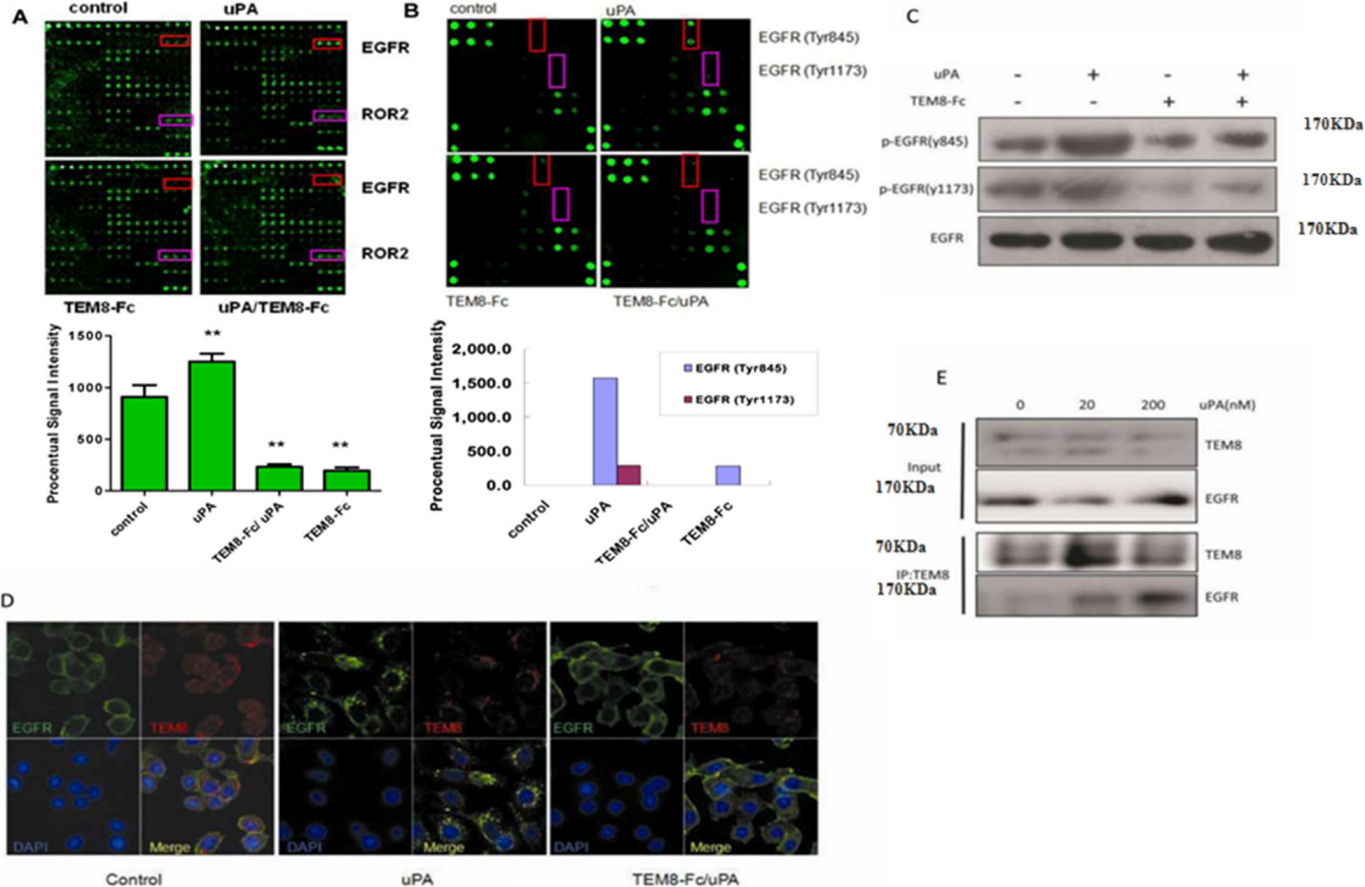
Induction of EGFR phosphorylation by uPA. **a** Treatment of HepG2 cells with uPA stimulates the phosphorylation of EGFR, which is blocked by TEM8-Fc, as detected by RayBio® Human RTK Phosphorylation Antibody Array 1. The fluorescent image (upper panel) and the quantification of that image (lower panel) are shown. **b** Treatment of HepG2 cells with uPA stimulates the phosphorylation of EGFR at Tyr845 and Tyr1173 as detected by RayBio® Human EGFR Phosphorylation Antibody Array 1. The fluorescent image (upper panel) and the quantification of that image (lower panel) are shown. **c** HepG2 cells were treated with uPA and/or TEM8-Fc as described in Materials and Methods, and then the cell lysates were subjected to immunoblotting with the indicated antibodies. **d** HepG2 cells were treated with uPA and/or TEM8-Fc, and then dual-color immunofluorescence was performed to determine the cellular localization of TEM8 and EGFR. **e** HepG2 cells were treated with uPA. The cell lysates were immunoprecipitated by TEM8 antibody and then subjected to immunoblotting with EGFR antibody. TEM8 proteins were knockdown in HepG2 cells and the cell lysates were then immunoblotted with the indicated antibodies

In order to investigate which of the classical signaling pathways could be activated following TEM8 phosphorylation, a phosphorylation antibody array simultaneously investigating relative phosphorylation states of 71 different human receptor tyrosine kinases (RTKs) was performed. The distribution of the human RTKs in the array is displayed in Additional file 1: Figure S4. As demonstrated in Fig. 6a, incubation of HepG2 cells with LMW-uPA significantly increased the EGFR phosphorylation (*p* = 0.024), and TEM8-Fc could antagonize this effect to a significant extent.

Since LMW-uPA significantly increased the EGFR phosphorylation, an EGFR phosphorylation antibody array consisting of seventeen EGFR phosphorylation sites (Additional file 1: Figure S5) was employed to determine which EGFR phosphorylation sites were affected. In the presence of LMW-uPA, phosphorylation of the classic EGFR target residues, EGFR Tyr845 and Tyr1173, were significantly increased (Fig. 6b). TEM8-Fc could also antagonize the effect of LMW-uPA. Consistent with these results, immunoblotting also showed that LMW-uPA increased EGFR Tyr845 and Tyr1173 phosphorylation, which was inhibited by TEM8-Fc treatment (Fig. 6c).

Interestingly, incubation with LMW-uPA caused TEM8 and EGFR to aggregate and co-localize on the cell surfaces, as demonstrated by immunofluorescence (Fig. 6d). The result of the IP experiment also showed that TEM8 could interact with EGFR under the presence of LMW-uPA, which had a dose dependent effect (Fig. 6e). Since EGFR is an important molecule for targeting cancer therapy, simultaneous blockade of TEM8 and EGFR may potentially have more potent anti-cancer effects.

### The TEM8/uPA interaction is important for tumor growth and metastasis

**Fig. 7:**
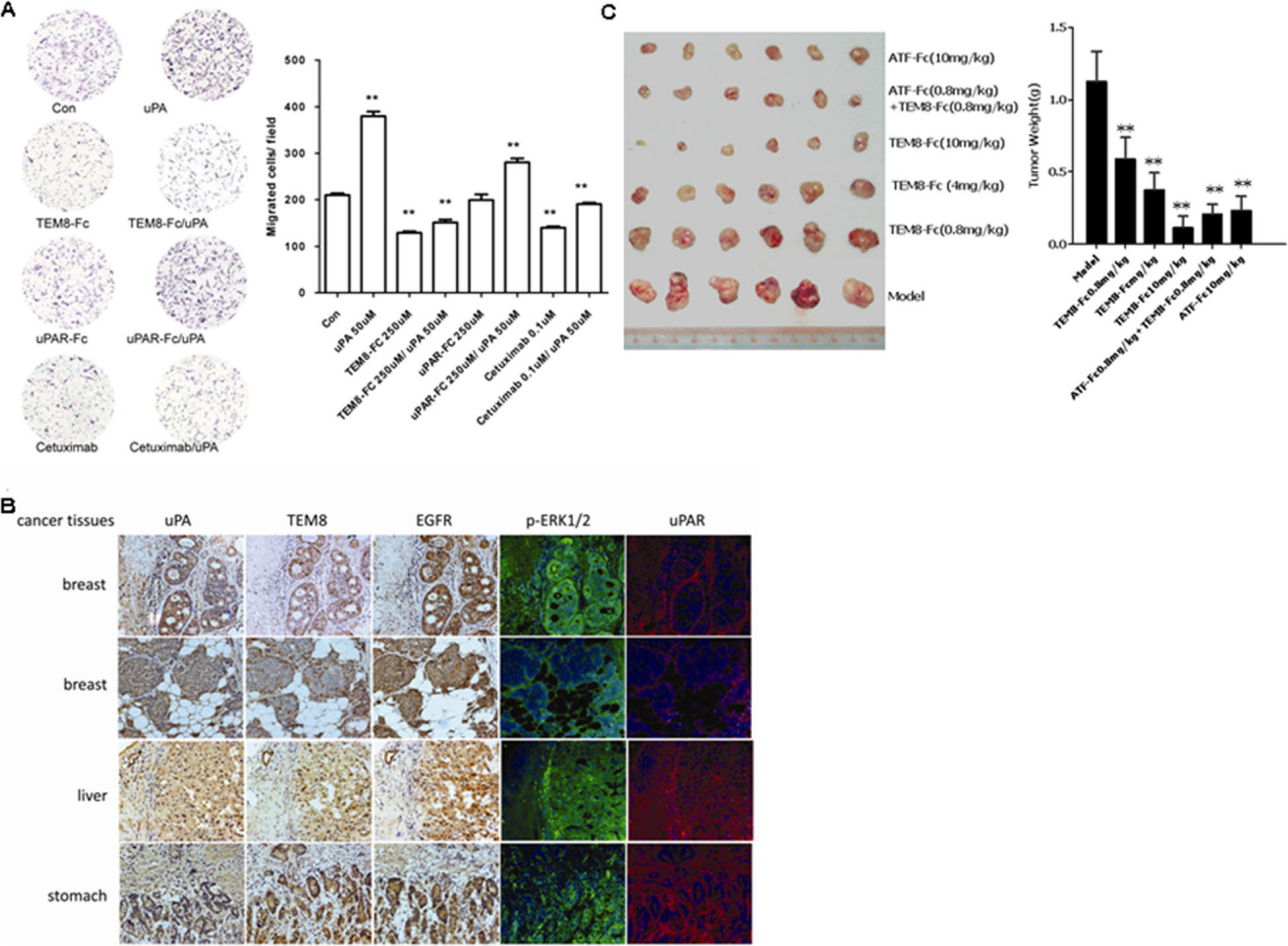
Effect of uPA/TEM8 interaction on tumor growth and metastasis. **a** For the migration assay, HepG2 cells (1 × 10^5^ cells/well) were seeded into the upper chambers of Transwell plates coated with 3 μg/ml collagen. Cells were stimulated with or without LMW-uPA in the presence or absence of TEM8-Fc for 4 h. Migrated cells were quantified as described in Materials and Methods. Similar results were obtained in three independent experiments; “a” indicates *p* < 0.001 vs. control group; “b” indicates *p* < 0.001 vs. LMW-uPA group. **b** The expression of uPA, TEM8, EGFR and uPAR and the ERK1/2 phosphorylation were examined on serial frozen cancer tissue sections by immunohistochemistry or immunofluorescence. **c** TEM8-Fc and ATF-Fc, at the indicated doses, substantially blocked the growth of subcutaneously implanted MCF-7 breast tumor cells in nude mice, when administered every 2 days for 3 weeks. * *p* < 0.01, ** *p* < 0.001 vs. control group. AF + TF refers to combined ATF-Fc and TEM8-Fc

**Fig. 8:**
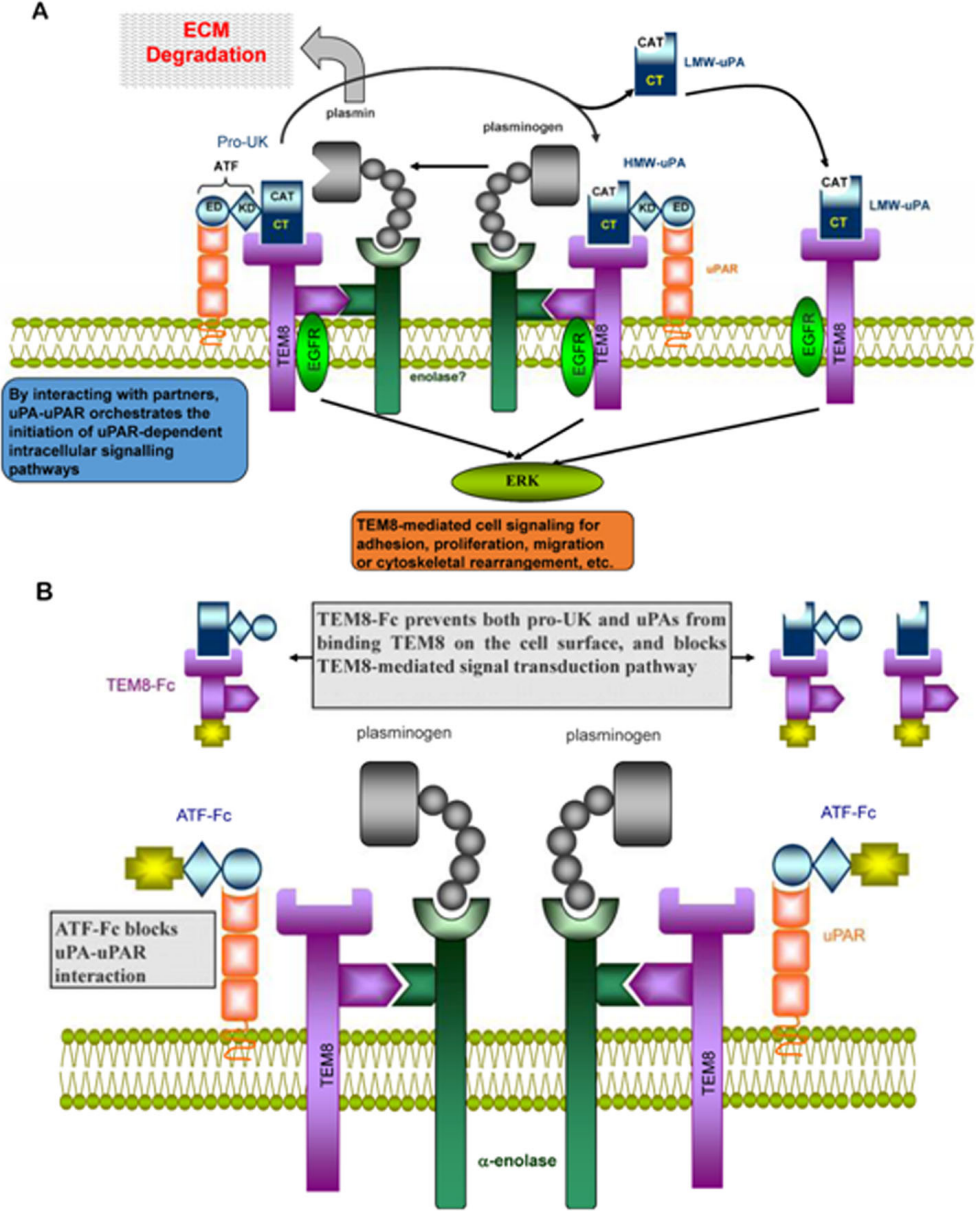
Schematic representation of the association between uPA and its binding partners. **a** The interaction of TEM8 with α-enolase brings uPA and plasminogen into close proximity, resulting in a highly efficient reciprocal activation of Pro-uPA/HMW-scuPA and plasminogen at the leading edge of the migrating cell, which then breaks down ECM components or activates latent growth factors. In addition, ligation of uPA to TEM8 may directly activate uPAR-independent intracellular signal transduction pathways (such as EGFR-ERK1/2) via the long cytoplasmic tail of TEM8. **b** ATF-Fc and TEM8-Fc completely block the cell surface-associated proteolytic activity of uPA as well as uPA-initiated cell signaling and cytoskeletal rearrangement

The role of the TEM8/uPA interaction in tumor growth and metastasis was investigated. To determine the role of TEM8 in uPA-induced cell migration, LMW-uPA lacking the uPAR binding domain was added to various tumor cells to stimulate migration. The results showed that LMW-uPA markedly increased the uPAR-independent migration of HepG2 hepatoma cells; this activity was inhibited significantly by TEM8-Fc (Fig. 7a). In addition, Cetuximab, an EGFR inhibitor used for the treatment of cancer, could also inhibited the uPA’s activity of stimulating migration, which further verified the LMW-uPA-induced cell migration was through EGFR-ERK1/2 pathway. Then we examined uPA, TEM8, EGFR and uPAR expression and the ERK1/2 phosphorylation on frozen cancer tissue sections by immunohistochemistry and immunofluorescence. We found all of uPA, TEM8 and EGFR were overexpressed and ERK1/2 was phosphorylated, and all of these above co-located on frozen breast cancer tissue sections. uPAR was also overexpressed, but it did not co-located with either the above. The same results were observed on the frozen cancer tissue sections of kidney, liver, stomach. These findings supported the notion that TEM8 and EGFR are co-expressed and interacted with each other under the presence of uPA in cancer cells and thus play crucial roles in tumor growth and metastasis in vivo (Fig. 7b). The potential therapeutic effect of TEM8-Fc on the growth of human tumors implanted subcutaneously in athymic nude mice was also investigated. As expected, TEM8-Fc significantly suppressed the growth and metastasis of the implanted tumors, and reversed the malignant phenotype of human xenografts. ATF-Fc is another antibody-like molecule targeting uPAR, which is generated by fusing the ATF portion of uPA to the Fc fragment of human IgG1. Administration of TEM8-Fc with ATF-Fc yielded synergistic anti-tumor activity (Fig. 7c). Therefore, as indicated in Fig. 8b, simultaneous blockade of the interactions between uPAR and TEM8 with uPA dramatically inhibited the cell surface-associated proteolytic activity of uPA as well as uPA-initiated cell signaling and/or cytoskeletal rearrangement, making this a most promising strategy for the treatment of a variety of solid tumors. Collectively, these data show that the uPA-TEM8 protein complex contributes substantially to the promotion of tumor growth and metastasis.

## Discussion

The data presented here show that uPA could be immunoprecipitated from a human malignant hepatoma tissue extract using TEM8-Fc. Purified uPA bound directly to TEM8-Fc with high affinity, in contrast to Herceptin, another Fc-containing protein. We also demonstrated that uPA can bind TEM8 at the cell surface and induce TEM8 phosphorylation, which is critical for uPA-induced tumor cell migration. These data indicate that TEM8 is a novel receptor for uPA. Binding of uPA to uPAR has been shown to play important roles in embryonic development, wound repair and tumor metastasis. However, multiple lines of evidence support the existence of other undefined receptors for uPA [23]. For example, uPA can activate cell-associated plasminogen, stimulate liver regeneration, and induce arteriogenesis and tumor cell migration in an uPAR-independent manner [24–28]. Therefore, identification of TEM8 as an uPA receptor contributes to our understanding of the biological functions of uPA. Since plasminogen activation plays a crucial role in tumor metastasis, and TEM8 expression is elevated in tumor endothelium, our findings reveal that TEM8 is a key molecule involved in tumorigenesis and progression.

Disruption of the *TEM8* gene in mice by targeted homologous recombination resulted in viable mice which reached adulthood without defects in physiological angiogenesis. However, histopathological analysis revealed an excess of ECM in several tissues, including the ovaries, uterus, skin and periodontal ligament of the incisors [29]. Interestingly, mutations in the TEM8 homologue, CMG2, have been found to cause juvenile hyaline fibromatosis and infantile systemic hyalinosis, disorders associated with the accumulation of amorphous, uncharacterized ECM [30, 31]. Trichrome staining of the affected tissues revealed the identity of the excess ECM as collagen; however, an increase in the number of fibroblasts was not evident [29]. Due to the fact that TEM8 has been found to bind collagen types I and VI in vitro [6, 8], in addition to uPA, as demonstrated here, we predicted that disruption of TEM8 could potentially lead to reduced degradation of these and other ECM proteins. These results suggest that both TEM8 and CMG2 play important roles in ECM homeostasis.

The finding that HMW-scuPA and LMW-uPA bind to TEM8 with a similar affinity indicates that the N-terminus of uPA is dispensable for the uPA-TEM8 interaction, which suggests that this interaction is distinct from the uPA-uPAR interaction. However, we found that TEM8 not only interacts with the LMW domain, but also the kringle domain of uPA. In this regard, the uPA-TEM8 interaction shares similarities with the interaction between uPA and integrin, since it has been reported that the kringle domain of uPA can directly interact with integrin alpha v beta 3 [32]. The binding does not affect the catalytic activity of uPA; therefore, a novel signal epitope (SE) should exist in the carboxyl-terminal region of uPA that mediates the uPA-TEM8 interaction. Although the precise mechanisms are still unclear, we speculate that ligation of uPA to TEM8 may initiate two important biological events simultaneously: degradation of pericellular matrix by activation of plasminogen, and induction of intrinsic chemotactic activity through the activation of several intracellular signal transduction pathways mediated by the complex cytoplasmic tail of TEM8 (as indicated in Fig. 8a). Both events are crucial to a variety of important pathophysiological processes, such as angiogenesis, embryonic development, and tumor invasion and/or metastasis. TEM8, as well as uPAR, localizes and concentrates uPA on the cell surface, increasing the efficiency of plasminogen activation, and subsequently potentiating plasmin-dependent degradation of the ECM. This creates an ideal pericellular environment for cell proliferation, migration and differentiation. In contrast, ligation of uPA to TEM8 through an independent SE in the carboxy-terminal region of uPA might directly activate proteolysis-independent intracellular signal transduction pathways via the long cytoplasmic tail of TEM8, which is distinct from the uPA-uPAR interaction. Intracellular signal transduction induced by the binding of uPA to uPAR requires the interaction of uPAR with other transmembrane partners, such as specific integrins, G-protein-coupled receptors and EGFR [15, 33], because uPAR lacks a cytoplasmic domain.

Laurence Abrami, et al. found when PA interacted with TEM8, the receptor could be phosphorylated, and then Src-like kinases were activated [14]. The human RTK Phosphorylation Antibody Array revealed that, after uPA bond TEM8 and induced TEM8 phosphorylation, uPA could specifically activate EGFR, and only increase the phosphorylation of the classic EGFR target residues Tyr845 and Tyr1173. These effects were completely abrogated by TEM8-Fc. Tyr-845 of EGFR has been thought to be the Src-dependent phosphorylation site [34], and Src Signaling is related to anthrax toxin endocytosis [14]. TEM8 may mediate uPA into the cells similar to the mechanism it mediate anthrax toxin. Similar interactions and signaling pathways with anthrax toxin endocytosis may occur in endothelial cells during angiogenesis. On the other hand, as described above,TEM8 shares high homology with integrins, and furthermore, it shares some interesting properties with integrins, though integrins are always being in the form of αβ dimmers [35]. It has been reports that one chain of the integrin binds actin could affect the interaction of the other chain for the ligand [36]. Similarly, TEM8 can interact with actin [9, 10, 12] and the interaction negatively regulates the binding of TEM8 for PA [9, 37]. To some extent, like CMG2, TEM8 can also be seen as a single chain of integrin [13]. It has been extensively documented that integrins can form a functional complex with EGFR and uPAR to mediate cell migration and angiogenesis [38], and our finding show that uPA can simultaneously induce TEM8 and EGFR phosphorylation. TEM8, as integrins, may also form a functional complex with EGFR and uPAR, when uPA simultaneously binds its receptors uPAR and TEM8 (binding two receptors at the same time can increase their affinity) in cell migration and angiogenesis, and then TEM8 and EGFR are simultaneously phosphorylated, which further induces downstream signaling molecules (such as ERK1/2 which was confirmed by Western Blotting) phosphorylation and cell migration and angiogenesis. Interestingly, uPA could induce TEM8 and EGFR co-localization at the cell surface, and TEM8-Fc completely blocked this effect. These data further support the view that TEM8 is the receptor of uPA.

In additional experiments, we have demonstrated that alpha enolase is another interacting partner of TEM8. Alpha enolase is a key glycolytic enzyme and also acts as a plasminogen receptor in a variety of cells, indicating that this enzyme plays an important role in some biological processes [39, 40]. The interaction between TEM8 and alpha enolase recruits uPA and plasminogen into close proximity with one another, resulting in a highly efficient proteolytic cascade at the cell surface. At the same time, limited proteolysis to the immediate pericellular environment protects surrounding tissue from damage (Fig. 8a).

In our previous study, we showed that TEM8 interacts with the M2 isoenzyme of pyruvate kinase [4]. It is very interesting to note that both pyruvate kinase and enolase are key enzymes in the glycolytic pathway. Therefore, we can suppose that local hypoxic conditions in tumor tissues lead to enhanced glycolysis, increased expression and extracellular release of pyruvate kinase, and increased expression and cell surface localization of enolase on tumor cells. Released pyruvate kinase will bind to TEM8 and stimulate new, localized blood vessel formation. Cell surface enolase links with TEM8, together with uPA and plasminogen, to form a highly efficient proteolytic cascade at the tumor cell surface, which promotes tumor metastasis. These processes supply sufficient nutrients to tumor cells and are crucial for rapid tumor growth. Taken together, we reveal that TEM8 is a novel receptor for uPA and is an attractive target for the development of new anti-cancer agents. In addition, just as VEGF-Trap blocks VEGF to suppress tumor growth and vascularization in vivo [41], TEM8-Fc may provide an intriguing anti-tumor agent, serving as a potent trap for uPA.

## Conclusions

Binding of uPA stimulates the phosphorylation of TEM8 and augments phosphorylation of EGFR and other signaling proteins. These demonstrate that TEM8 is a novel receptor for uPA,, which may play a significant role in the regulation of tumor growth and metastasis.and provide new insights to explain the uPAR-independent functions of uPA on many physiological and pathological events.

## Additional file

Additional file 1: **Figure S1.** Schematic diagram of three different forms of uPA. The three existing forms of uPA. **Figure S2.** Three different forms of recombinant uPA. SDS-PAGE analysis of HMW-tcuPA (lanes 1-3), LMW-uPA (lanes 4-6) and HMW-scuPA (lanes 7-9) under reducing and non-reducing conditions. **Figure S3.** LMW-uPA interacts with TEM8. HepG2 cells were cultured in 96-well plates, then fixed and treated with acidified buffer to remove endogenous uPA. LMW-uPA was added at the indicated concentrations in the presence of absence of 2000 nM TEM8-Fc, or 2000 nM Herceptin, and the cell surface-based fibrinolytic activity was measured. Data were expressed as mean±SD of triplicate wells. Similar results were obtained in independent experiments. **Figure S4.** RayBio® Human RTK Phosphorylation Antibody Array G-series 1 Map. The attribution from the phosphorylation to the different human Receptor Tyrosine Kinases was obtained with Figure S3, where 71 different human receptor tyrosine kinases (RTKs) were represented. Dots A1, B1, C1, D1, E1, F1, G1, H1, I1, M16, N16 and O16 were pos (positive controls) and A2, B2, C2, D2, E2, F2, G16, H16, I16, J16, K16 and L16 were neg (negative controls). Those dots ensured the accuracy of the results. **Figure S5.** RayBio® Human EGFR Phosphorylation Antibody Array G-series 1 Map. The attribution from the phosphorylation to the different specific sites for Human EGFR family was obtained with Figure S4, where 17 different specific sites were represented. Dots A1, B1, C1, A2, B2, C2, I7 and I8 were pos (positive controls) and E1, E2, G7 and G8 were neg (negative controls). Those dots ensured the accuracy of the results. **Table S1.** Combined MALDI and MALDI-QTOF data for identification of proteins in Figure 1. **Table S2.** Biacore kinetics and affinity results for binding of different uPAs to TEM8. a. N=3; b. ND, not determined. (DOC 6661 kb)

## Abbreviations

ANTXR1: Anthrax toxin receptor 1
CMG2: Capillary morphogenesis gene 2
HMW-scuPA: The single chain high molecular weight zymogen
HMW-tcuPA: The full-length two-chain high molecular weight urokinase-type plasminogen activator
LMW-uPA: The low molecular weight uPA enzyme
MIDAS: The metal ion-dependent adhesion site
PA: The protective antigen
RTKs: Human receptor tyrosine kinases
SPR: Surface plasmon resonance
TEM8: Tumor endothelial marker 8
TEM8-Fc: A recombinant fusion protein comprising the extracellular domain of human TEM8 linked to the Fc portion of human IgG1
uPA: Urokinase-type plasminogen activator
