# Supplementary material for: TEM8 functions as a receptor for uPA and mediates uPA-stimulated EGFR phosphorylation
Source: Cell Commun Signal. 2018 Sep 21;16:62. doi: 10.1186/s12964-018-0272-8 (PMC6151050; doi:10.1186/s12964-018-0272-8)

**Additional file 1**

Table S1: Combined MALDI and MALDI-QTOF data for identification of proteins in Figure 1.

| Protein name | MW | PI |  |  |
| --- | --- | --- | --- | --- |
| Sequence | Charge | | XC | DeltaCn |
| Plasminogen activator, urokinase | 51724.53 | 8.57 |  |  |
| DYSADTLAHHNDIALLK | 2 |  | 4.7389 | 0.5874 |
| FEVENLILHK | 2 |  | 2.665 | 0.3295 |
| IIGGEFTTIENQPWFAAIYR | 2 |  | 5.7761 | 0.6219 |
| KEDYIVYLGR | 2 |  | 3.5503 | 0.4534 |
| MTLTGIVSWGR | 2 |  | 2.7322 | 0.3928 |
| TDSCQGDSGGPLVCSLQGR | 2 |  | 4.8555 | 0.5967 |
| YFSNIHWCNCPK | 2 |  | 3.1025 | 0.7111 |

**Table S2: Biacore kinetics and affinity results for binding of different uPAs to TEM8 ^a^.**

| protein | Mn^2+^ in the buffer (mM) | k_a_ (1/Ms) | k_d_ (1/s) | *K*_D_ (M) |
| --- | --- | --- | --- | --- |
| PA | 0 | ND^b^ | ND | ND |
| PA | 2 | 5.78×10^4^±4.25×10^3^ | 4.18×10^-4^±2.17×10^-6^ | 7.22×10^-9^±5.47×10^-9^ |
| HMW-scuPA | 0 | 1.56×10^4^±6.38×10^3^ | 2.25×10^-4^±4.94×10^-5^ | 1.44×10^-8^±8.28×10^-9^ |
| HMW-scuPA | 2 | 3.25×10^4^±2.71×10^3^ | 5.40×10^-4^±9.15×10^-5^ | 1.66×10^-8^±9.72×10^-8^ |
| HMW-tcuPA | 0 | 5.86×10^3^±5.64×10^2^ | 4.53×10^-4^±8.73×10^-6^ | 7.73×10^-8^±5.37×10^-9^ |
| HMW-tcuPA | 2 | 6.43×10^4^±7.73×10^3^ | 1.91×10^-3^±9.28×10^-4^ | 2.97×10^-8^±6.17×10^-8^ |
| LMW-uPA | 0 | 23.3±3.2 | 2.99×10^-4^±3.43×10^-6^ | 1.28×10^-5^±8.39×10^-6^ |
| LMW-uPA | 2 | 2.07×10^4^±2.18×10^2^ | 6.45×10^-4^±3.70×10^-5^ | 3.11×10^-8^±2.90×10^-9^ |
| BSA | 2 | ND | ND | ND |

1. N=3 b. ND, not determined.

**Supplemental Experimental Procedures**

**Antibodies and reagents**

The protein A Sepharose 4 Fast Flow affinity column was purchased from Amersham Biosciences, Uppsala, Sweden; Streamline™ rProtein A was purchased from GE Healthcare, Uppsala, Sweden; Herceptin was obtained from Roche, Grenzach-Wyhlen, Germany; PA was from Merck, Darmstadt, Germany; protease inhibitor cocktail, type I collagen and chromogenic urokinase substrate S-2444 were purchased from Sigma, St Louis, MO, USA; Lipofectamine™ 2000 reagent was from Gibco BRL, Grand Island, NY, USA; rabbit IgG and protein A/G-Sepharose were from Santa Cruz Biotechnology, Inc., Santa Cruz, CA, USA; antibodies recognizing RFP and GFP were purchased from MBL, Nagoya, Japan; anti-uPA, anti-uPAR and anti-TEM8 (ab15724) antibodies were from Abcam Ltd, Cambridge, UK; anti-phospho-EGFR (Tyr845), anti-phospho-EGFR (Tyr1173), anti-phospho- ERK1/2 and anti-phosphotyrosine (p-Tyr100) antibodies were from Cell Signaling Technology, Inc., Beverly, MA, USA; horseradish peroxidase-conjugated secondary antibodies (anti-rabbit IgG, anti-goat IgG, anti-mouse IgG and anti-human IgG) and anti-actin were from Beijing Zhongshan Jinqiao Biotechnology Ltd., Beijing, China.

**Analysis of binding of TEM8 to recombinant uPA by ELISA**

Interaction of the TEM8-Fc fusion protein with uPA was examined by direct ELISA. To do this, 96-well plates were coated with 0.1 μg of recombinant PA, HMW-scuPA, commercially available high molecular weight two-chain urokinase-type plasminogen activator (termed as HMW-tcuPA, Techpool Biochemical Pharmaceutical Ltd. Guangzhou, China), or LMW-uPA in Tris-buffered saline (TBS) for 16 h at 4°C. The plates were washed three times with TBST (0.05% Tween-20 in TBS), and blocked with 100 µl/well of blocking buffer (3% BSA in TBS) for 1 hour at room temperature. After three washes, 0-250 ng of purified TEM8-Fc or Herceptin in 100 µl diluting buffer (3% BSA, 0.05% Tween-20 in TBS) in the presence of 2 mM MnCl_2_, was added to the wells and incubated at 37°C for 1 hour. Following three washes, the samples were incubated with 100 µl/well of diluted HRP conjugated goat anti-human IgG (1:20,000) for 1 hour at 37°C. The plates were washed and 100 µl of developing solution (3,3’,5,5’-Tetramethylbenzidine/H_2_O_2_) was added. After 15 minutes, stop solution (2M H_2_SO_4_) was added and the plates were analyzed on a microplate reader at a wavelength of 450 nm. The results were calculated as mean absorbance (A_450nm_) of triplicate wells for each sample.

**Additional file 1**

**Figure S1: schematic diagram of three different forms of uPA.** The three existing forms of uPA.

**Figure S2: Three different forms of recombinant uPA.** SDS-PAGE analysis of HMW-tcuPA (lanes 1-3), LMW-uPA (lanes 4-6) and HMW-scuPA (lanes 7-9) under reducing and non-reducing conditions.

**Figure S3: LMW-uPA interacts with TEM8.** HepG2 cells were cultured in 96-well plates, then fixed and treated with acidified buffer to remove endogenous uPA. LMW-uPA was added at the indicated concentrations in the presence of absence of 2000 nM TEM8-Fc, or 2000 nM Herceptin, and the cell surface-based fibrinolytic activity was measured. Data were expressed as mean±SD of triplicate wells. Similar results were obtained in independent experiments.

**Figure S4: RayBio® Human RTK Phosphorylation Antibody Array G-series 1 Map.** The attribution from the phosphorylation to the different human Receptor Tyrosine Kinases was obtained with Figure S3, where 71 different human receptor tyrosine kinases (RTKs) were represented. Dots A1, B1, C1, D1, E1, F1, G1, H1, I1, M16, N16 and O16 were pos (positive controls) and A2, B2, C2, D2, E2, F2, G16, H16, I16, J16, K16 and L16 were neg (negative controls). Those dots ensured the accuracy of the results.

**Figure S5: RayBio® Human EGFR Phosphorylation Antibody Array G-series 1 Map.** The attribution from the phosphorylation to the different specific sites for Human EGFR family was obtained with Figure S4, where 17 different specific sites were represented. Dots A1, B1, C1, A2, B2, C2, I7 and I8 were pos (positive controls) and E1, E2, G7 and G8 were neg (negative controls). Those dots ensured the accuracy of the results.

**Supplementary Figures**


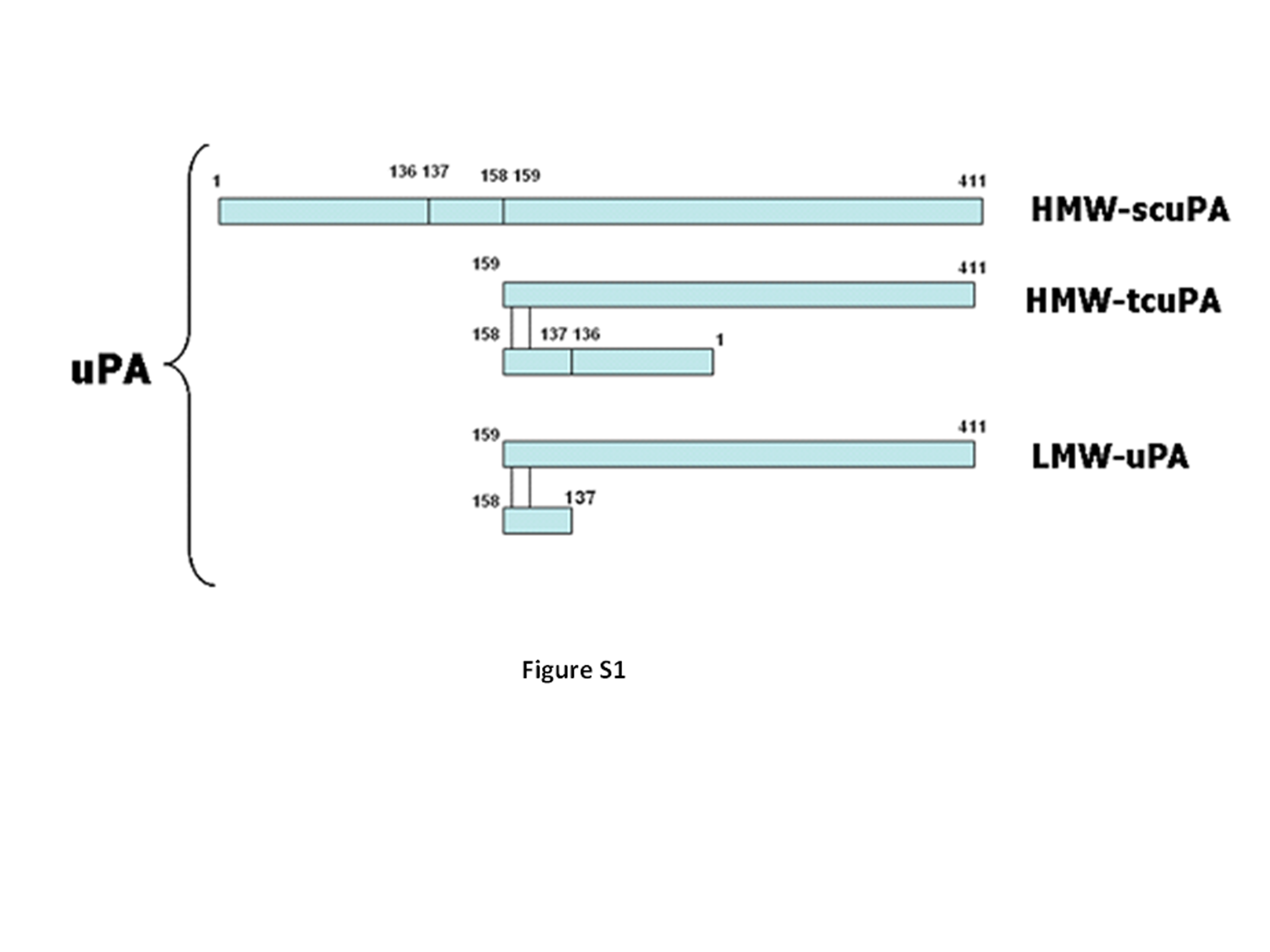


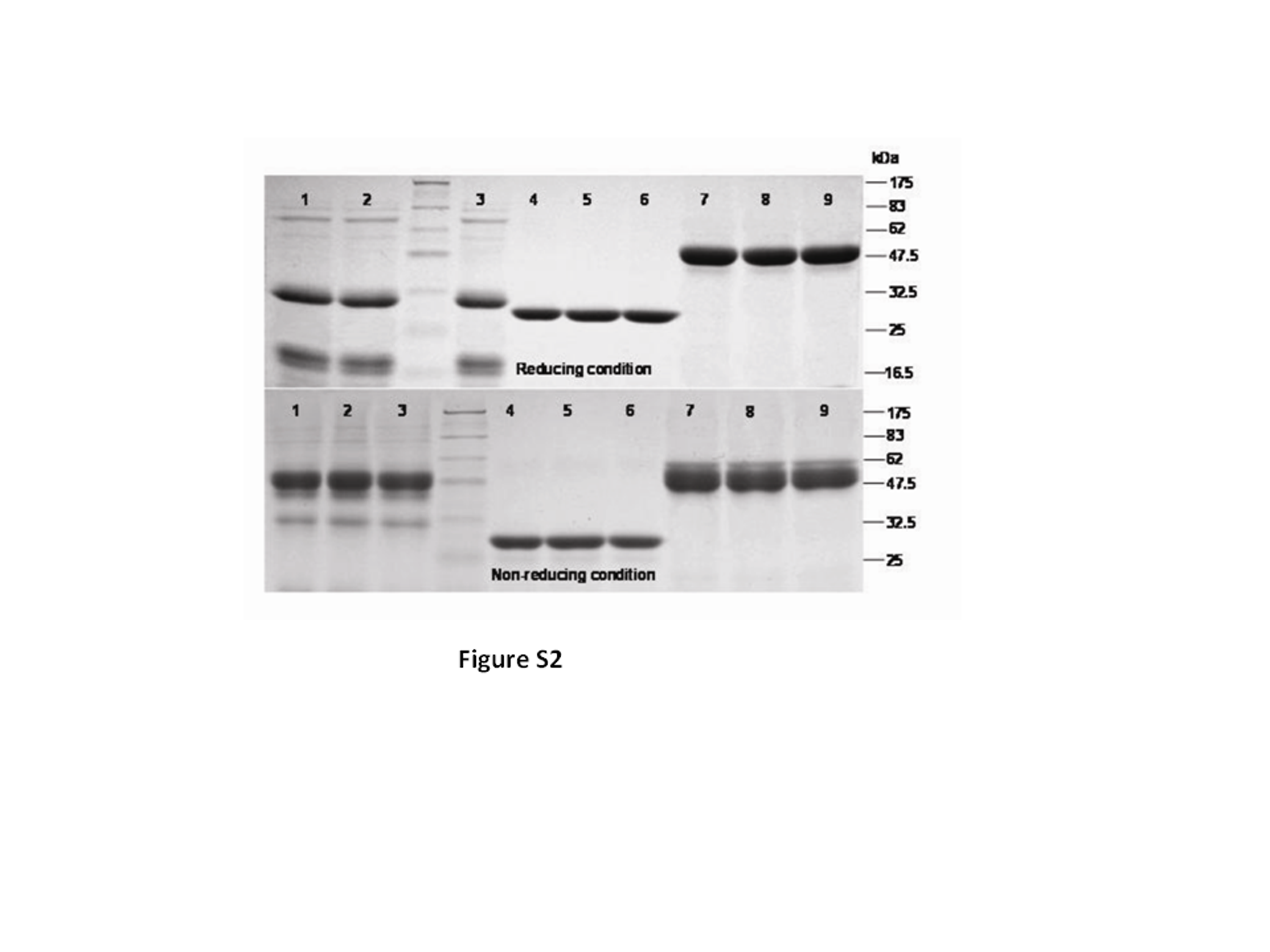


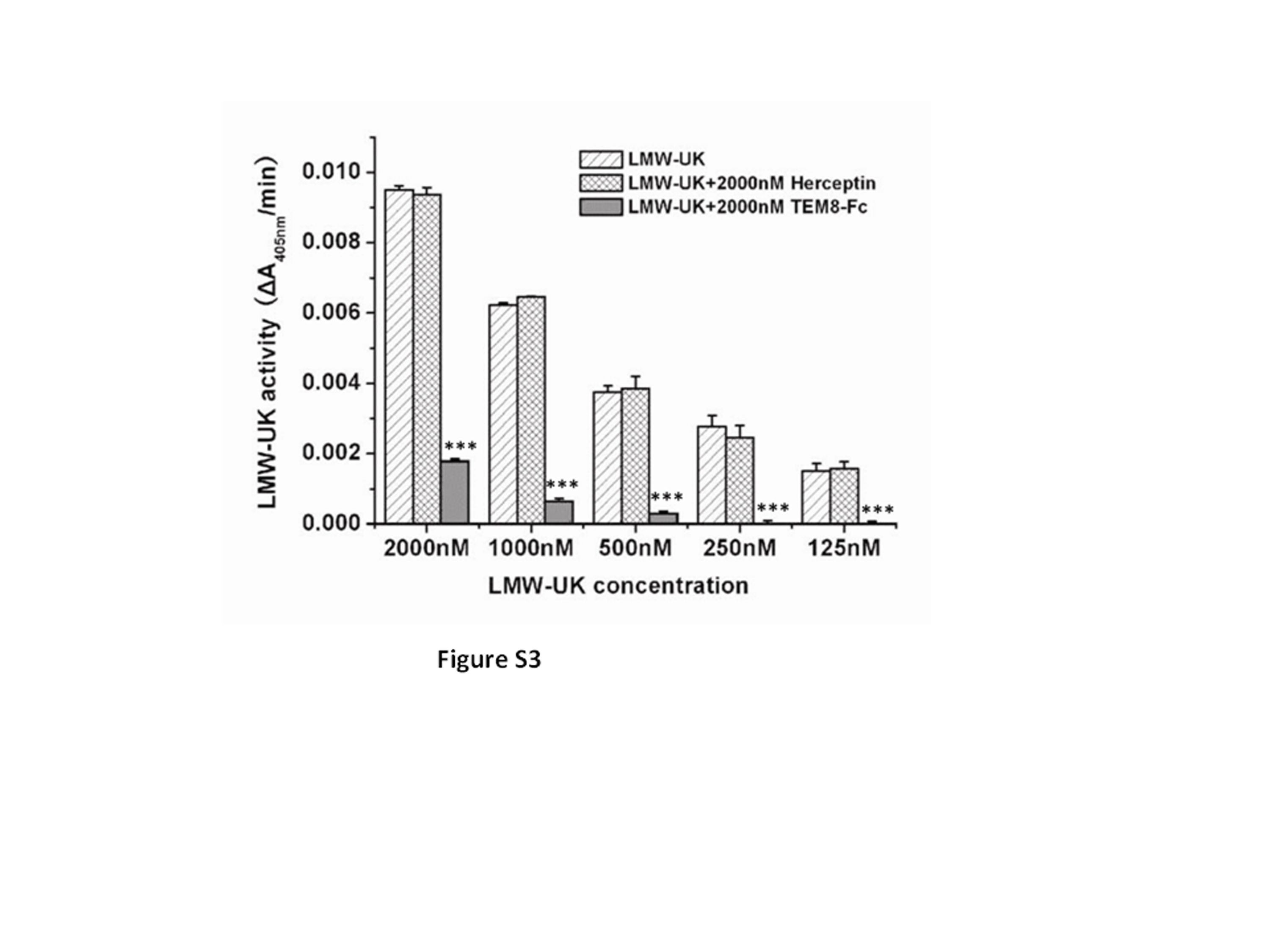


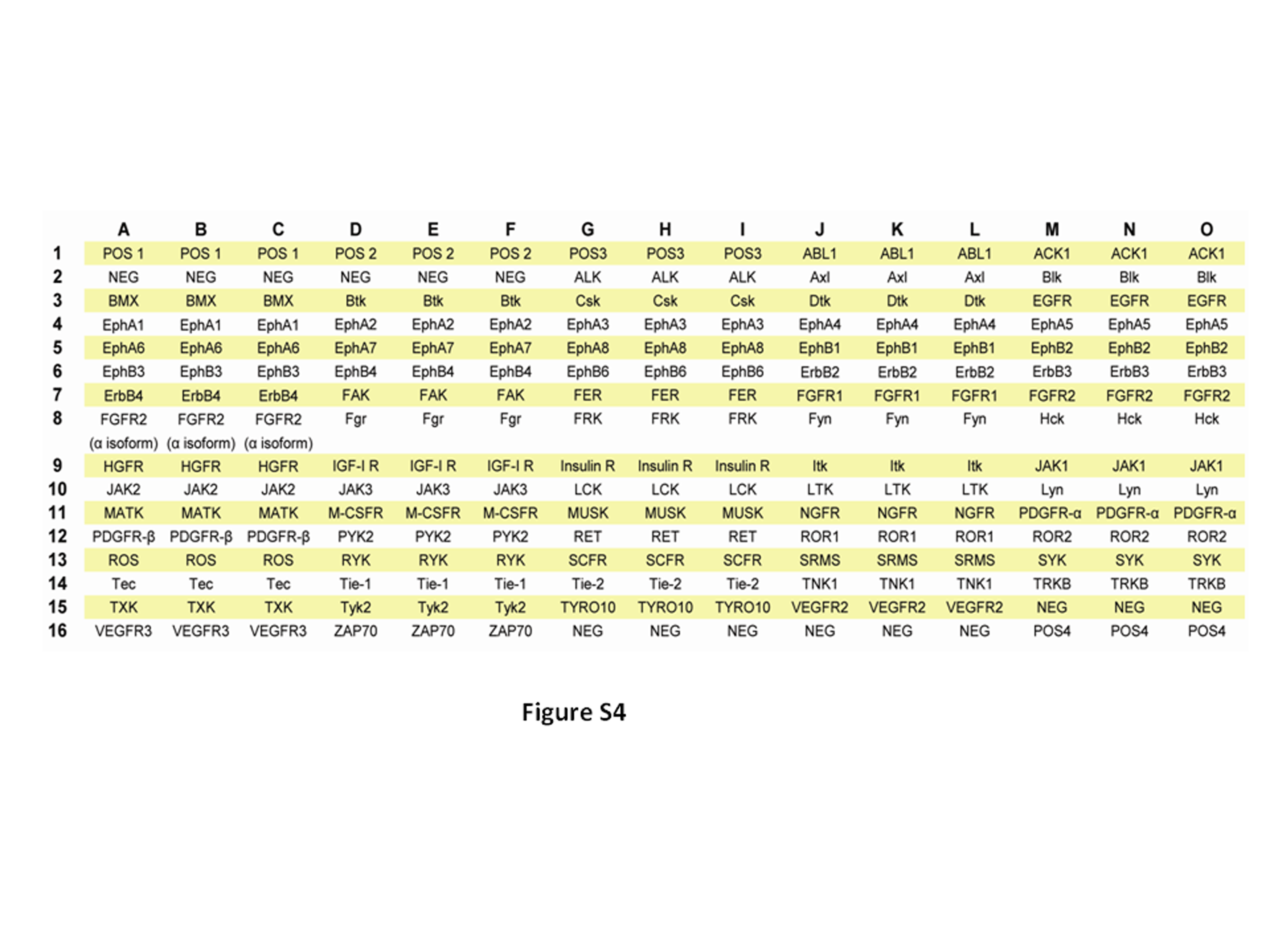


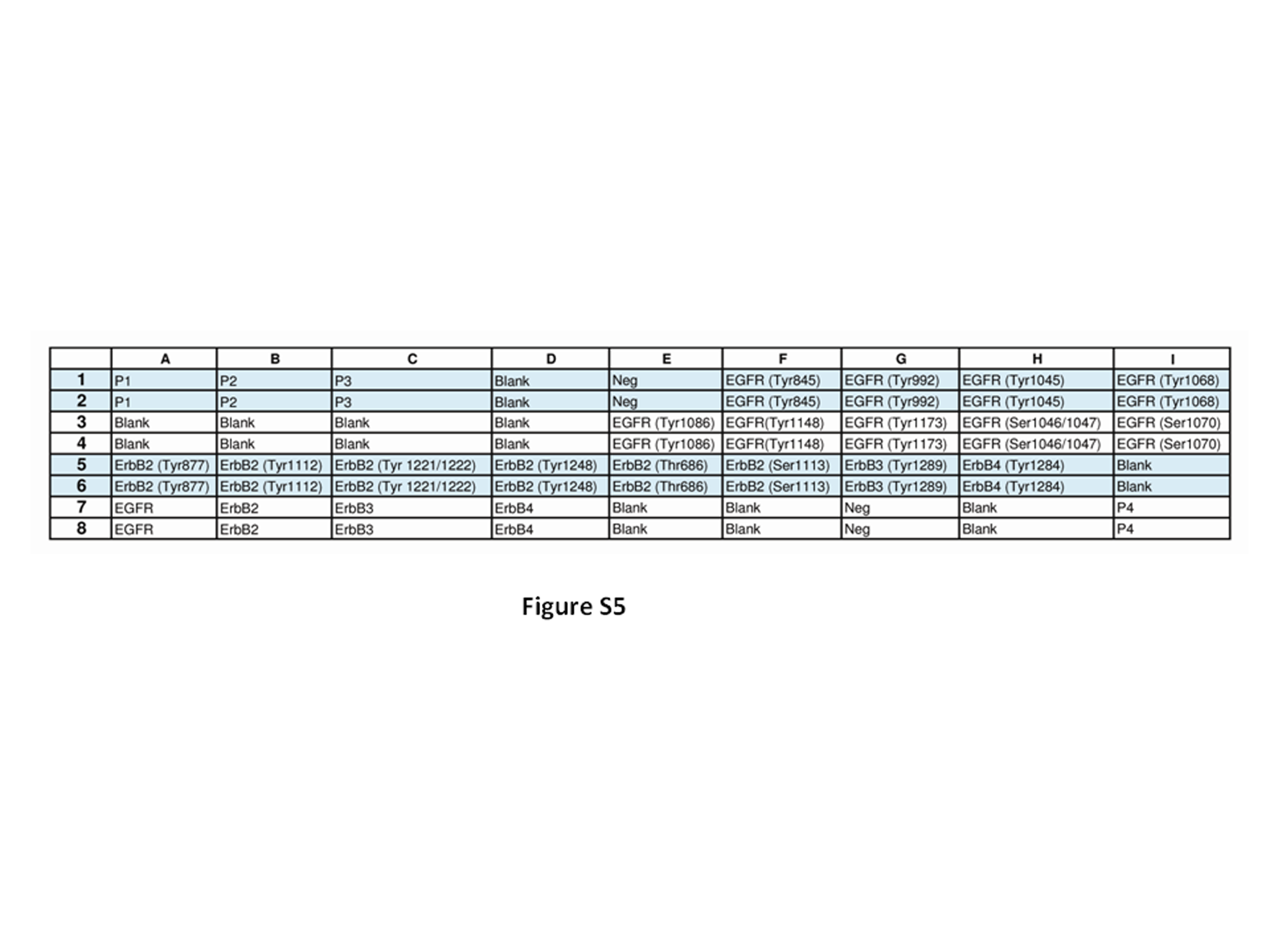

Supplement: Supplementary file 1 — Figure S1. Schematic diagram of three different forms of uPA. The three existing forms of uPA. Figure S2. Three different forms of recombinant uPA. SDS-PAGE analysis of HMW-tcuPA (lanes 1-3), LMW-uPA (lanes 4-6) and HMW-scuPA (lanes 7-9) under reducing and non-reducing conditions. Figure S3. LMW-uPA interacts with TEM8. HepG2 cells were cultured in 96-well plates, then fixed and treated with acidified buffer to remove endogenous uPA. LMW-uPA was added at the indicated concentrations in the presence of absence of 2000 nM TEM8-Fc, or 2000 nM Herceptin, and the cell surface-based fibrinolytic activity was measured. Data were expressed as mean±SD of triplicate wells. Similar results were obtained in independent experiments. Figure S4. RayBio® Human RTK Phosphorylation Antibody Array G-series 1 Map. The attribution from the phosphorylation to the different human Receptor Tyrosine Kinases was obtained with Figure S3, where 71 different human receptor tyrosine kinases (RTKs) were represented. Dots A1, B1, C1, D1, E1, F1, G1, H1, I1, M16, N16 and O16 were pos (positive controls) and A2, B2, C2, D2, E2, F2, G16, H16, I16, J16, K16 and L16 were neg (negative controls). Those dots ensured the accuracy of the results. Figure S5. RayBio® Human EGFR Phosphorylation Antibody Array G-series 1 Map. The attribution from the phosphorylation to the different specific sites for Human EGFR family was obtained with Figure S4, where 17 different specific sites were represented. Dots A1, B1, C1, A2, B2, C2, I7 and I8 were pos (positive controls) and E1, E2, G7 and G8 were neg (negative controls). Those dots ensured the accuracy of the results. Table S1. Combined MALDI and MALDI-QTOF data for identification of proteins in Figure 1. Table S2. Biacore kinetics and affinity results for binding of different uPAs to TEM8. a. N=3; b. ND, not determined. (DOC 6661 kb) [file 12964_2018_272_MOESM1_ESM.doc]
